# Intrinsic Dynamics of Amorphous Ice Revealed by a Heterodyne Signal in X-ray Photon Correlation Spectroscopy Experiments

**DOI:** 10.1021/acs.jpclett.3c02470

**Published:** 2023-12-01

**Authors:** Hailong Li, Marjorie Ladd-Parada, Aigerim Karina, Francesco Dallari, Mario Reiser, Fivos Perakis, Nele N. Striker, Michael Sprung, Fabian Westermeier, Gerhard Grübel, Werner Steffen, Felix Lehmkühler, Katrin Amann-Winkel

**Affiliations:** † Max-Planck-Institute for Polymer Research, Ackermannweg 10, 55128 Mainz, Germany; ‡ State Key Laboratory of Fine Chemicals, School of Chemical Engineering, Dalian University of Technology, Dalian 116024, China; § Department of Physics, AlbaNova University Center, Stockholm University, Roslagstullsbacken 21, SE-10691 Stockholm, Sweden; ∥ Department of Chemistry, KTH Royal Institute of Technology, Roslagstullsbacken 21, 11421 Stockholm, Sweden; ⊥ Deutsches Elektronen-Synchrotron DESY, Notkestrasse 85, 22607 Hamburg, Germany; # Hamburg Centre for Ultrafast Imaging, Luruper Chaussee 149, 22761 Hamburg, Germany; 7 European X-ray Free-Electron Laser, Holzkoppel 4, 22869 Schenefeld, Germany; 8 Institute of Physics, Johannes Gutenberg University Mainz, Staudingerweg 7, 55128 Mainz, Germany

## Abstract

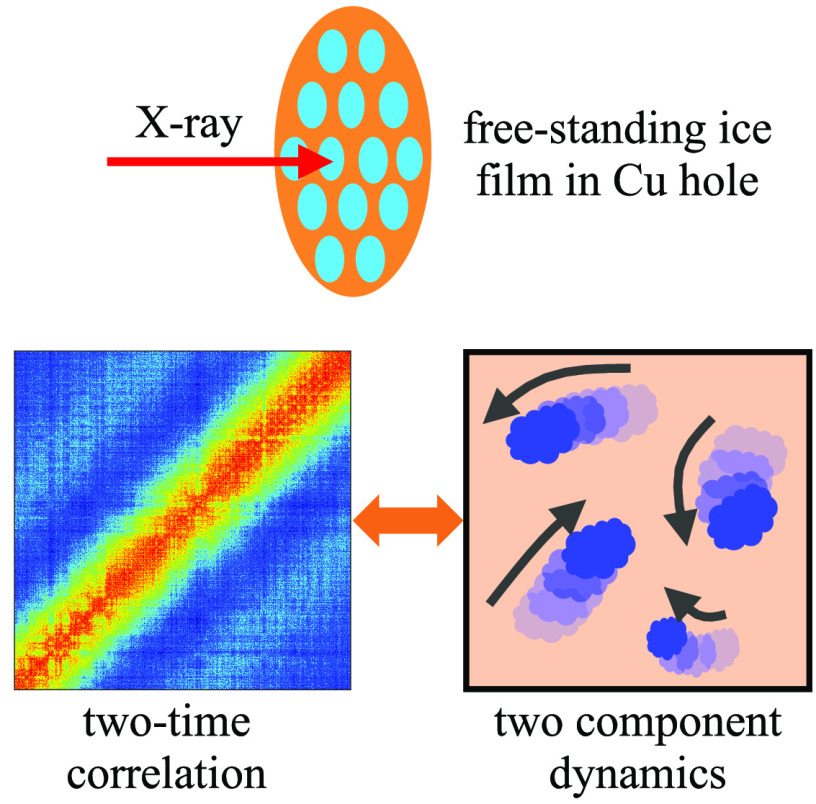

Unraveling the mechanism of water’s glass transition and the interconnection between amorphous ices and liquid water plays an important role in our overall understanding of water. X-ray photon correlation spectroscopy (XPCS) experiments were conducted to study the dynamics and the complex interplay between the hypothesized glass transition in high-density amorphous ice (HDA) and the subsequent transition to low-density amorphous ice (LDA). Our XPCS experiments demonstrate that a heterodyne signal appears in the correlation function. Such a signal is known to originate from the interplay of a static component and a dynamic component. Quantitative analysis was performed on this heterodyne signal to extract the intrinsic dynamics of amorphous ice during the HDA–LDA transition. An angular dependence indicates non-isotropic, heterogeneous dynamics in the sample. Using the Stokes–Einstein relation to extract diffusion coefficients, the data are consistent with the scenario of static LDA islands floating within a diffusive matrix of high-density liquid water.

Water is the key component for the existence of life on Earth and plays a central role in a wide range of scientific disciplines such as biology, chemistry, atmospheric chemistry, geophysics, food science, and electrocatalysis. It is also one of the most unusual liquids with many anomalous properties, such as the density maximum at 4 °C, a lower density in the solid phase than in the liquid phase, and many more.^1,2^ The existence of two liquid phases with different densities [high- and low-density liquid (HDL and LDL, respectively)] has been proposed to explain the origin of the anomalous properties of water.^3−7^ The two liquid states are thought to be the counterparts of high- and low-density amorphous ices (HDA and LDA,^1,8,9^ respectively). Recently, amorphous ice’s glassy nature has been challenged by experimental findings on fast compression pathways that suggest that HDA is not connected to a liquid state but rather a disordered crystalline state.^10^ On the contrary, current high-pressure studies on the very-high-density (VHDA) state show that both VHDA and HDA are connected to an ultraviscous liquid at high pressure but reach the same HDL state at intermediate pressures of <0.3 GPa.^11^ The dynamic and structural nature of amorphous ice is still controversial.^10−12^

As of today, X-ray photon correlation spectroscopy (XPCS) has been used as a powerful technique to investigate dynamics of different systems, like colloidal^13−18^ and polymeric^19−25^ materials, in wide- and small-angle scattering (WAXS and SAXS, respectively) regimes. Similar in principle to dynamic light scattering,^26^ XPCS may readily be used to probe structural dynamics throughout different length scales from atomic to hundreds of nanometers. Most of these studies are focused on the dynamics of systems in the equilibrium state or under different flow conditions, where no phase transition or structural change happens. For example, the advective and diffusive dynamics of colloidal particle dispersion was studied under homogeneous shear flow.^27^ However, physiochemical processes often also happen under non-equilibrium conditions or involve phase transitions. Few dynamic studies have focused on phase transition or phase ordering processes. For example, the dynamics of a protein solution during liquid–liquid phase separation upon low-temperature quenches was determined and revealed distinctly different dynamical regimes.^28^ Heterogeneous dynamics during the transformation of the face-centered cubic phase to the hexagonal close-packed phase of cobalt was investigated.^29^ Recently, the dynamics and structural changes of amorphous ice during the HDA–LDA transition were tracked by combining wide-angle X-ray scattering (WAXS) and XPCS at small scattering angles (SAXS).^4^ Two dynamic processes were found during the transition; one was related to a liquid-like motion. The nature of the second process, however, remained unclear.

Here, we study the phase transition of amorphous ices but use a distinct preparation method for ice samples. Instead of a powdered sample, we use a compact, free-standing piece of ice, which is 10 times thinner than that in the previous study.^4^ We now observe a previously hidden heterodyne signal in a one-component system, which allows us to shed light on the nucleation and growth process during the phase transition from HDA to LDA.^5,30−33^

Upon warming, HDA transforms into LDA through an exothermic transition^32^ accompanied by a volume change of 20%.^33^Figure 1a shows five representative two-dimensional (2D) WAXS patterns recorded at different temperatures, starting from equilibrated HDA (eHDA) at 90 K to LDA at 120 K (*T*_cryostat_). The corresponding azimuthally integrated intensities *I*(*Q*) in the momentum transfer range of 1.3 Å^–1^ < *Q* < 3.0 Å^–1^ are plotted in Figure 1b. The characteristic diffraction maximum of eHDA at *Q* = 2.17 Å^–1^ was observed at temperatures of ≤90 K. At 100 K, a weak shoulder of LDA at *Q* = 1.7 Å^–1^ appears, and its intensity increases with temperature. Simultaneously, the scattering intensity recorded in SAXS geometry increases between 90 and 115 K (see Figure S1 for details). Note that the reported temperatures are not measured directly at the sample position; therefore, we expect the actual sample temperature to be slightly higher [*T*_cryostat_ + 5 K (see the Supporting Information)]. The sample was held for 30 min at the desired temperature, before the measurement was begun. The bimodal scattering intensity in the WAXS pattern implies phase coexistence of eHDA and LDA and is consistent with our previous results.^4,30,34^ The complete transition to LDA happens above 120 K (*T*_sample_ ≈ 125 K). This is slightly lower than the values from other studies of eHDA^30,35^ but can be explained by a different sample preparation (see also the Supporting Information). We note that intense Bragg spots can be found on the scattering patterns (Figure 1a). These spots appear as sharp Bragg peaks from crystalline ice in our WAXS curves (Figure 1b). We attribute them to the presence of a small amount of hexagonal ice that condensed on the sample during sample transfer.^34^ As the amount of these hexagonal ices is expected to be small and not embedded in the high-pressure ice matrix, it is reasonable to neglect its effect on the dynamics.

**Figure 1:**
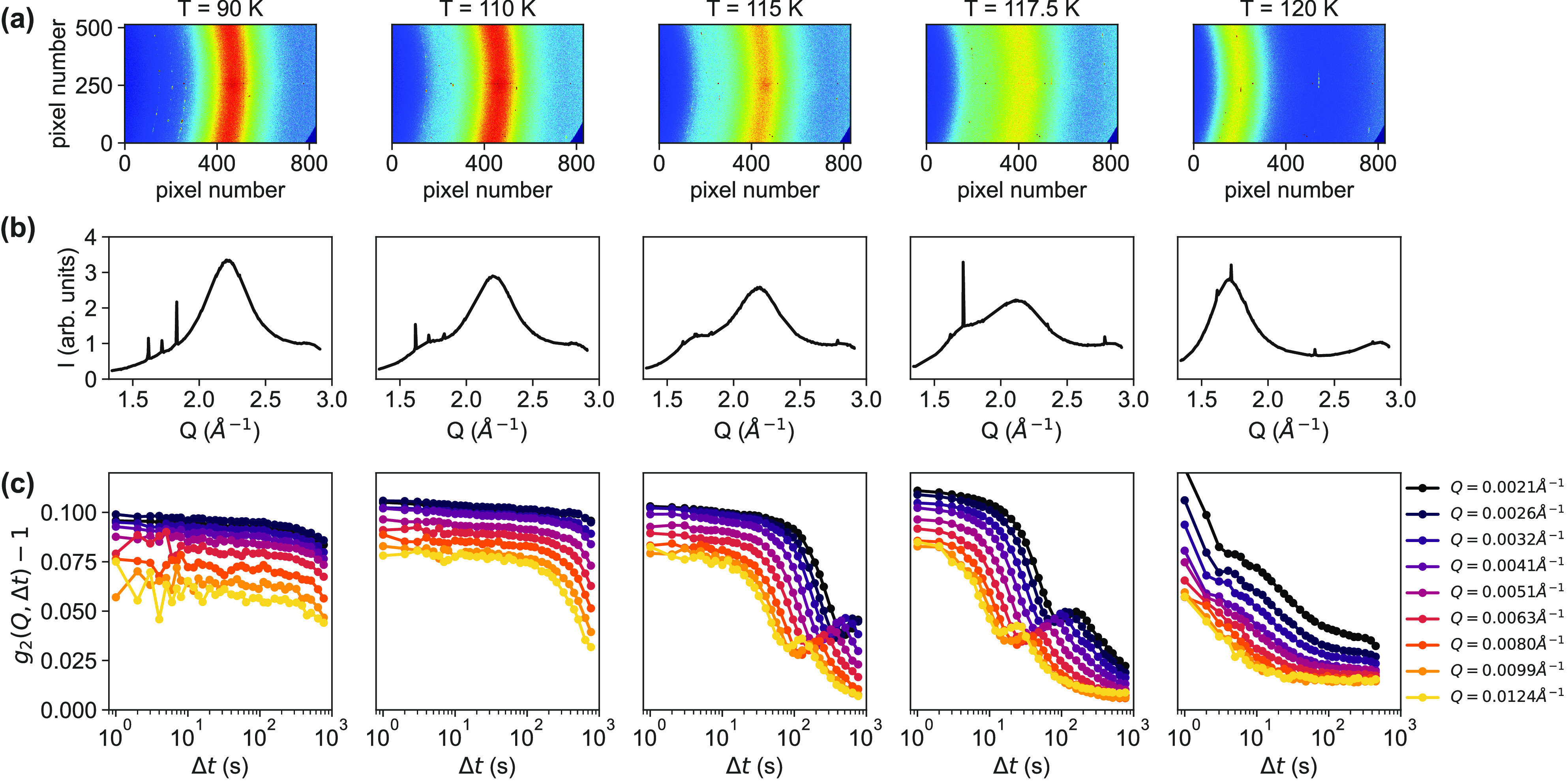
*In situ* WAXS/XPCS experiments using two detectors simultaneously. (a) Representative 2D WAXS patterns recorded at different temperatures. Note that all given temperatures refer to the measured *T*_cryostat_; the sample temperature is estimated to be 5 K higher. (b) Corresponding azimuthally integrated intensity *I*(*Q*) in the momentum transfer range of 1.3 Å^–1^ < *Q* < 3.0 Å^–1^. Details can be found in the Supporting Information. (c) Intensity autocorrelation functions *g*_2_(*Q*, Δ*t*) – 1 calculated at different values of *Q* for the corresponding temperatures.

Figure 1c shows the calculated intensity autocorrelation functions *g*_2_(*Q*, Δ*t*) at different values of *Q* and temperatures. For all temperatures, the speckle contrast slightly decreases with an increase in *Q*. This is seen as the short time plateau value of the correlation function decreases. Such an effect was also observed in other studies^13,28,36,37^ and might be related to a faster decay process that could not be resolved in the experiment. From the decay of speckle contrast, we determined the length scale at which such a localized process occurs to be ∼45 Å (see Figure S2 for details). Upon heating to 120 K, it is evident that the dynamics become faster. Surprisingly, an additional oscillation was observed in the *g*_2_ curves at 115 and 117.5 K. This phenomenon was not observed previously and might have been hidden in the powder samples.^4^ The oscillation may be explained by heterodyne mixing of a diffusive and static component. We discuss this in the following.

To investigate whether the sample was in equilibrium during the measurements, we additionally calculate the two-time correlation function (TTC),^38^ which is defined as
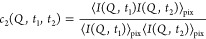
1where *I*(*Q*, *t*_1_) and *I*(*Q*, *t*_2_) denote the intensity of a pixel at distinct times *t*_1_ and *t*_2_, respectively. The subscript “pix” implies that the averaging is, in this case, solely performed over pixels within the same *Q*-bin and not over time. The TTCs for temperatures ranging from 90 to 120 K at *Q* = 0.0032 Å^–1^ are shown in Figure 2. A narrow intensity on the diagonal line for higher temperatures (115, 117.5, and 120 K) indicates rather fast dynamics, while a broad and smeared out intensity marks “slower” dynamics, as the correlations perpendicular to the diagonal line decay more slowly. Additionally, the oscillatory behavior is visible for temperatures of 115 and 117.5 K, which is consistent with the results depicted in Figure 1c. The TTC line shape for all temperatures reveals that the sample is stable over time and does not exhibit a change in dynamics during the measurements.

**Figure 2:**
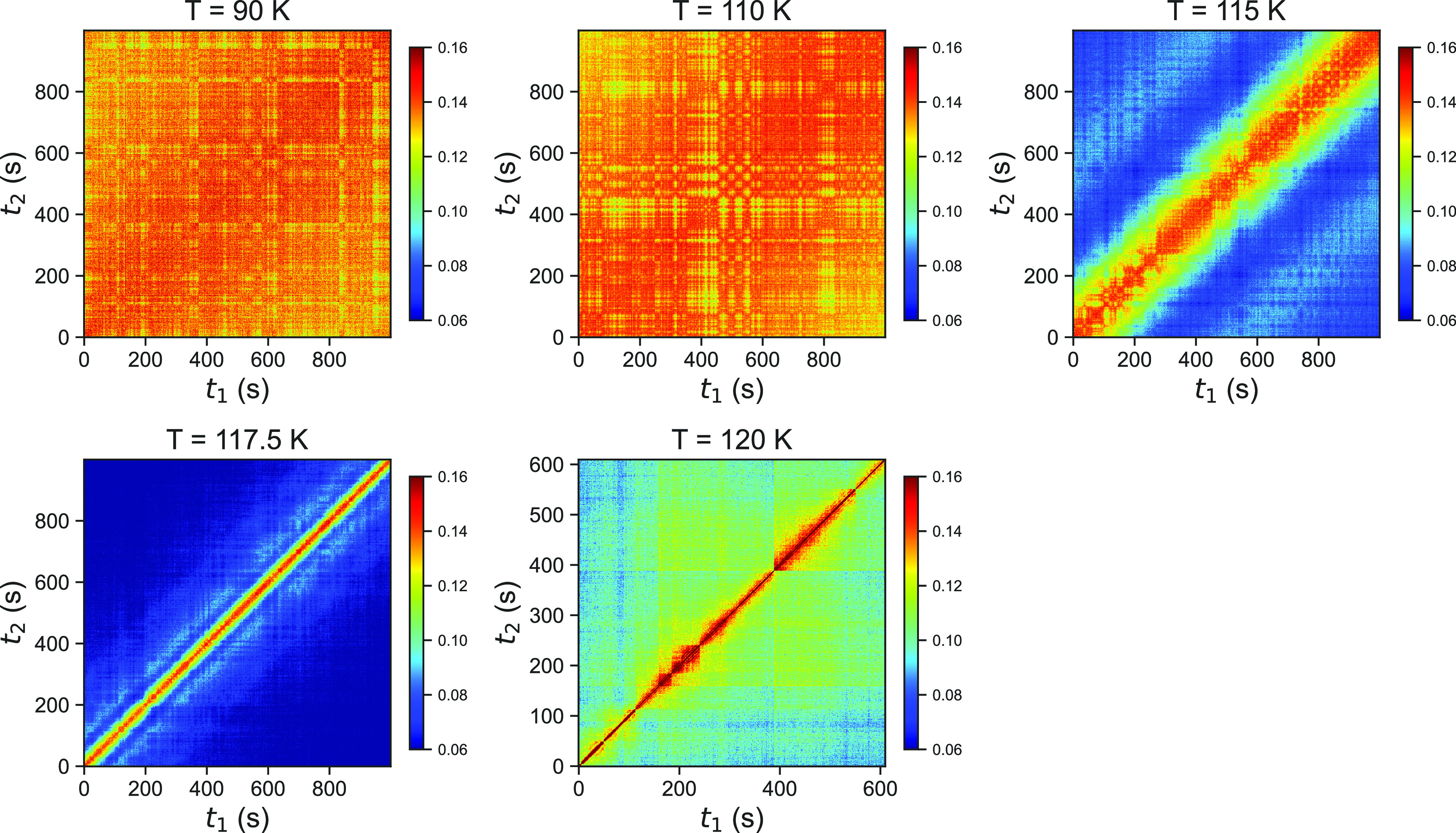
Two-time correlation function (TTC) at different temperatures indicated in the panel tiles at momentum transfer *Q* = 0.0032 Å^–1^. Note that at 120 K the data were collected for only ∼600 s. All given temperatures refer to the measured *T*_cryostat_; the sample temperature is estimated to be 5 K higher.

To quantitatively evaluate the dynamics at the different temperatures, in particular, once approaching the state of coexistence, a fit to the *g*_2_ curves including the oscillations is necessary. Although a sum of Kohlrausch–Williams–Watts (KWW) functions^39^ is successfully applied for systems with multiple dynamics,^4,28^ it is not suitable for our case (see Figure S3 for details). We applied different models to our data, including shear flow as reported in the literature,^27,40^ but noticed that none of them could describe the data (see Figures S4 and S5 for details). However, a master equation can be used to describe the decorrelation of a two-component system.^41−44^ Inspired by this idea, we obtain the following intensity–intensity autocorrelation function:
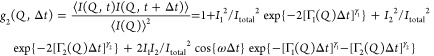
2where *I*(*Q*, *t*) is the intensity at the modulus of the momentum transfer *Q* = 4π sin(θ)/λ, Δ*t* denotes the correlation time delay, the brackets ⟨···⟩ indicate averaging over all detector pixels within the same *Q* interval as well as all frames at different times *t*, *I*_1_ and *I*_2_ are the scattering intensities of the two different components, *I*_total_ is the total scattering intensity, Γ_1_(*Q*) and Γ_2_(*Q*) are the *Q*-dependent relaxation rates (1/τ_1_ and 1/τ_2_), ω is the oscillation frequency and relates to the heterodyne period (2π/ω), and γ_1_ and γ_2_ are the Kohlrausch–Williams–Watts (KWW) exponents. Stretched correlation functions (γ < 1) are typically found in supercooled liquids, while Brownian diffusive processes are characterized by a γ = 1. Glasses usually show compressed exponentials (γ > 1), but for different reasons (stress relaxation). The heterodyne equation (eq 2) does not require necessarily that either of the dynamical processes be diffusive. Only if the relaxation rate Γ(*Q*) has a linear relationship with *Q*^2^, namely Γ(*Q*) = *D*_0_*Q*^2^, is a diffusive motion present, where *D*_0_ represents the Stokes–Einstein diffusion coefficient.^19,36^

Equation 2 was fitted to all of the data in this work. Panels a and d of Figure 3 represent the fitting results for the *g*_2_ curves at *Q* = 0.0099 Å^–1^ for 115 and 117.5 K, respectively. More fitting results are displayed in Figures S6 and S7. The contribution of the first exponential part *I*_1_^2^ exp{−2[Γ_1_(*Q*)Δ*t*]^γ_1_^} in eq 2 to the total *g*_2_ curve is small (blue dashed curve in panels a and d of Figure 3). The *Q* dependence of 1/τ_1_ (Figure S14) at two different temperatures indicates that the fast decay process is very robust and undergoes a hyperdiffusion or ballistic motion, which would be related to γ_1_ = 2. We therefore kept γ_1_ = 2 as a constant value for all of the later fitting procedures. However, we noticed that the fitting results would not be affected by changing the value for γ_1_ to 1, as the contribution of this part to the total *g*_2_ is small. In the following, we focus on the dominant second exponential part *I*_2_^2^ exp{−2[Γ_2_(*Q*)Δ*t*]^γ_2_^} and the third oscillatory part. Panels b and e of Figure 3 summarize the characteristic times obtained from the fitting of the second exponential component (black dashed curve in panels a and d of Figure 3) at different *Q* values at 115 and 117.5 K, respectively. An almost *Q*^2^ dependence of the extracted relaxation rates is observed with an additional small offset.^34^ The green solid line depicts the result of a fit 1/τ = *D*_0_*Q*^2^ + *c* with a diffusion coefficients (*D*_0_) of 12 and 63 Å^2^/s for 115 and 117.5 K, respectively, and an offset *c* of 0.0004 s^–1^ for both temperatures. A linear Q dependence 1/τ ∼ *Q* (red dashed–dotted line) and a power law fit 1/τ ∼ *Q*^*p*^ (blue solid line) are plotted for comparison but fail to describe the data. It is evident that the dynamics become faster and diffusive when 117.5 K is approached. This is accompanied by an increasing velocity of the sample changing from 2.9 to 14.5 Å/s, which is extracted from a linear fit of the heterodyne period 2π/ω as a function of *Q*^–1^ (green solid line in panels c and f of Figure 3, left *y*-axis). We further investigate the KWW exponent, γ_2_, of the second exponential process. Except for the last four data points at small values of *Q*, at 115 K, which have large error bars due to limited statistics, we observed that γ_2_ is ∼1 (red dashed line in Figure 3c). In contrast, when the temperature is increased to 117.5 K, γ_2_ becomes <1 (Figure 3f, blue axis); hence, a crossover to a stretched exponential relaxation behavior with a strong *Q* dependence takes place. This broad distribution of relaxation times is not in full agreement with the finding of a diffusive liquid-like behavior.^4^ Moreover, γ_2_ decreases with an increase in *Q*, indicating that the liquid is more rigid at smaller length scales. We further investigated potential heterogeneities by calculating *g*_2_ for different azimuthal directions.

**Figure 3:**
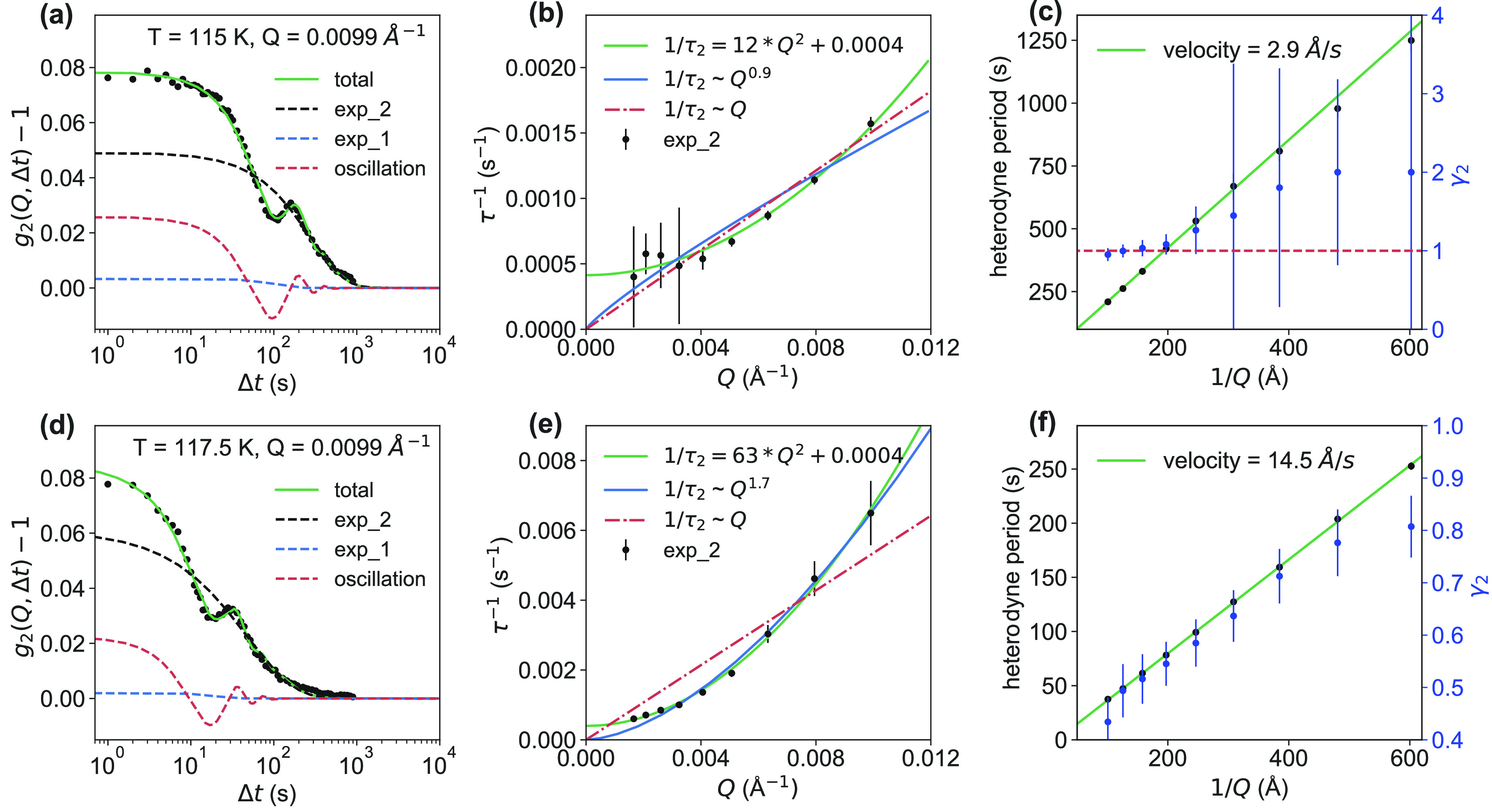
Quantitative analysis of the *g*_2_ curves. (a and d) Representatives of experimental data (black dots) showing oscillations obtained at *Q* = 0.0099 Å^–1^ for 115 and 117.5 K (*T*_cryostat_), respectively. The data are fitted with eq 2. The green solid, black dashed, blue dashed, and red dashed lines indicate the total, first and second exponential components, and oscillatory component of the model, respectively. (b and e) Characteristic time obtained from fitting of the second exponential component at different *Q* values at 115 and 117.5 K, respectively. (c and f) Heterodyne period 2π/ω and linear fit (green solid line) to calculate the velocity (left axis). KWW exponent γ_2_ of the second exponential component (blue, right axis).

We additionally calculated the *g*_2_ functions at different azimuthal angles for each temperature and *Q* as depicted in Figure 4a, hence now treating *Q* as a vector. *g*_2_ curves at different values of ϕ for 115 K and *Q* = 0.0041 Å^–1^ are plotted in Figure 4b, and appearing streaks have been masked out before integration. The *g*_2_ curves are shifted vertically for the sake of clarity. More results can be found in Figures S9, S10, and S12. The observed oscillation occurs at different times for different ϕ angles. For some specific angles, like 45° and 225°, the oscillations are no longer visible. The same fitting procedure (eq 2) was performed on all of the *g*_2_ curves at different *Q* values, angles, and temperatures. We determined the velocity by fitting the heterodyne period as a function of *Q*^–1^, as shown in the example in Figure 4c, where a velocity of 13.7 Å/s was extracted for 270° at 117.5 K (Figure 4c). Figure 4d summarizes the velocities at different angles for sample A at 115 and 117.5 K and sample B (two positions) at 117.5 and 120 K. The maximum velocity was observed at ∼135° and ∼315° for both temperatures and both samples, and the minimum velocity was observed orthogonal from the angle of the velocity maximum. The angular dependence of the velocity, and therefore also the heterodyning, shows a similar trend in different samples. A similar behavior of the angle-dependent velocity was reported by Dallari et al.^45^ when they investigated the stress relaxation in repulsive colloidal glasses. As a coincidence, in our measurements streaks appear on the detector (Figures S9 and S10) at ∼45°. Streaks have also been observed in other directions in other samples and temperatures, but here they appear mostly at this angle. We assume that the streaks are related to grain boundaries or stress.^44^ This is, the reason for the angle-dependent velocity observed here is most probably related to the heterogeneity of the macroscopic morphology inside the free-standing amorphous ice film during the sample preparation process. From optical microscopy images,^34^ it is known that the sample contains cracks and macroscopic grain boundaries. More detailed studies are required to fully understand this phenomenon. However, we can exclude a gravitational flow due to the sample characteristics, and lateral flow, which in other experiments^27,40^ has been used to describe the oscillations, fails to describe our data (see Figures S4 and S5). We also exclude the possibility of the signal originating from surface roughness, as concluded from the ratio between factors *I*_1_ and *I*_2_ from eq 2 in Figure S8.

**Figure 4:**
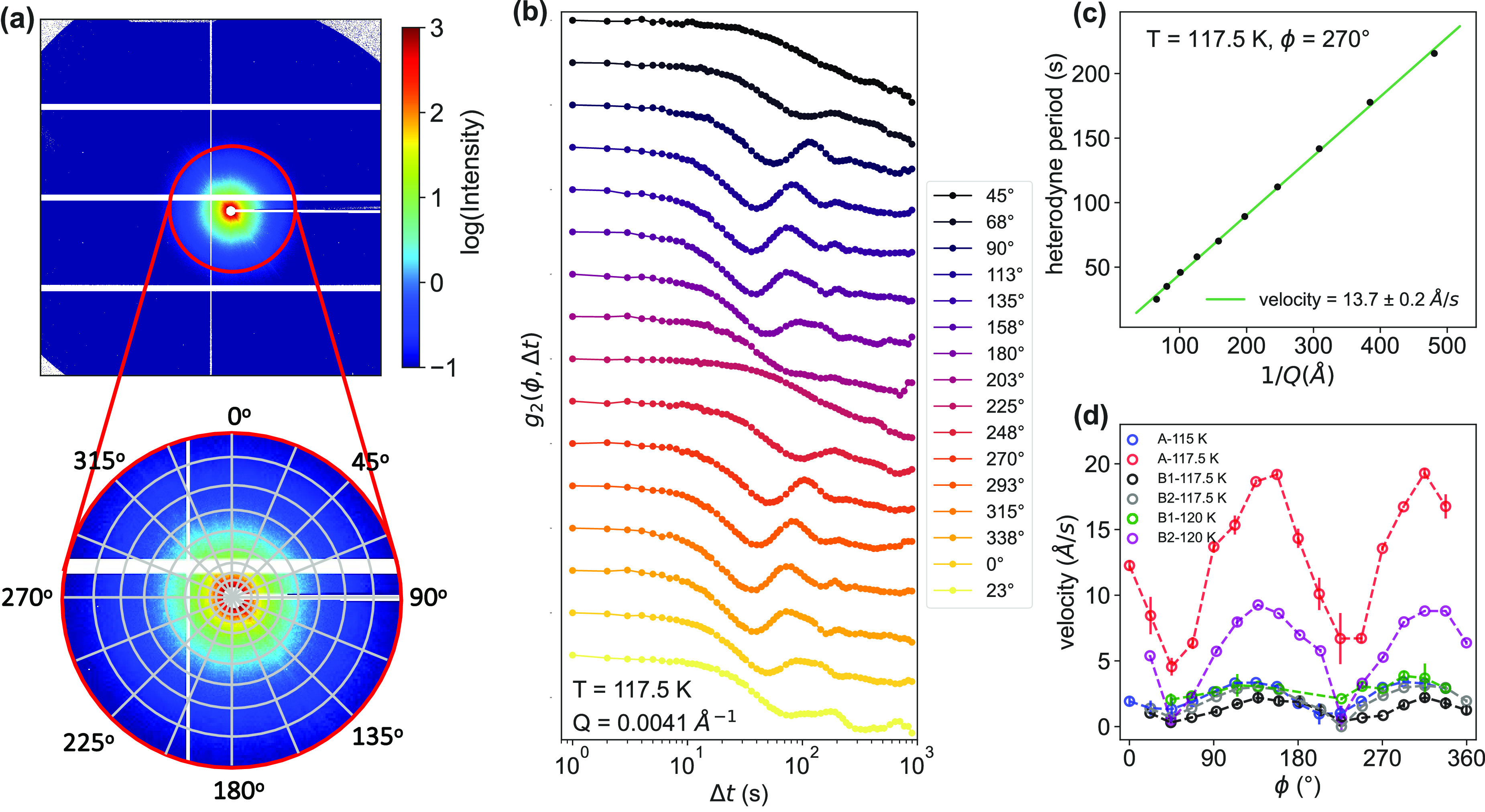
Angle-dependent dynamics. (a) 2D small-angle X-ray scattering pattern. The radial/azimuthal bins indicate the regions where the correlation functions of the individual pixels were averaged. (b) Intensity autocorrelation functions *g*_2_(*Q*, Δ*t*) calculated for different angles at 117.5 K and *Q* = 0.0041 Å^–1^. The *g*_2_ curves are shifted vertically for the sake of clarity. (c) Heterodyne period 2π/ω as a function of *Q*^–1^ at 117.5 K and ϕ = 270°. From the linear fit (green solid line), a velocity of the sample of 13.7 Å/s is obtained. (d) Velocity as a function of azimuthal angle ϕ for sample A at 115 and 117.5 K and sample B (two positions) at 117.5 and 120 K. Note that all given temperatures refer to the measured *T*_cryostat_; the sample temperature is estimated to be 5 K higher.

An oscillatory behavior of the *g*_2_ curves is typically observed in a heterodyne detection scheme of dynamic light scattering (DLS)^46^ and XPCS,^25,47−49^ where an external static reference must be applied. The phase shift in the scattering due to the motion of the sample with respect to the fixed reference yields the oscillation. However, in this work, the oscillatory behavior was observed in a homodyne detection scheme without adding any external static reference. This phenomenon is rare but has been observed in flowing media, such as sedimentation.^50−52^ A similar oscillatory behavior was discovered by Gutt et al. two decades ago using XPCS in reflection geometry,^53^ where they investigated the capillary waves on liquid water in grazing-incidence small-angle X-ray scattering (GISAXS) geometry. This heterodyne XPCS signal in reflection geometry was later extended to understand the surface dynamics of thin films during sputter deposition by Ulbrandt et al.^54^ and Ju et al.^55^ They demonstrated that the variable mixing of surface and bulk scattering signals causes oscillation, which suggests that an external static reference is not necessary for heterodyne detection. This idea was supported by the homodyne DLS work in supramolecular self-assembly of triarylamine molecules with tailored side groups by the Buhler group,^56,57^ where a similar oscillation behavior was observed and was attributed to two components in the system: a dangling fiber and microgel with different dynamics. Inspired by the work of Ulbrandt et al.^54^ and Buhler et al.,^56,57^ we propose that the oscillatory behavior (heterodyne signal) observed in our work is due to the coexistence of two components, which have different dynamics and distinct glass transition temperatures.^32^ The two components could be HDL and LDA, as the oscillations appear simultaneously with the observed transformation in the WAXS signal. Calorimetry and dielectric spectroscopy suggest the glass transition onset temperature to be *T*_g_ (eHDA) = 115 K and *T*_g_ (LDA) = 136 K, which is consistent with the previous XPCS data on powder samples.^4^ The measurements presented here are taken below the glass transition temperature of LDA. Some sample spots also show crystalline ice peaks (Figure S13) at the temperatures at which the oscillatory component is observed, which suggests that the heterodyne mixing could also be related to HDL and crystalline ice. Interestingly, this oscillatory behavior was not found for the powder amorphous ice samples,^4^ which might be caused by the bulk sample being confined between two diamond windows, thus not allowing for a fast expansion. Alternatively, the oscillations might be hidden by other effects caused by the grainy structure of the powdered bulk samples. In the work presented here, the XPCS experiments were performed on free-standing amorphous ice films, which allows for a macroscopic expansion with a volume change of 20% during the HDA–LDA transition, which is another possible source of the heterodyne signal. We ensure annealing of the sample for 20 min before each measurement, hence allowing thermally activated motion to relax; after 20 min, the WAXS signal remains stable. Still, it is a metastable system, and a fraction of water molecules will, also after equilibration, be displaced when high-density domains expand to low-density regions. The velocity obtained from the heterodyne signal can be related to this displacement (expansion). The liquid-like motion of the high-density matrix and the expansion to low-density are two correlated processes that are difficult to disentangle; the heterodyne signal now allows a first estimation. The free-standing amorphous ice film provides us with a new model sample system for investigating the intrinsic dynamics of the glass transition of amorphous ice’s glass transition.

Our previous data from eHDA-grid samples^34^ show that eHDA enters a hyperdiffusive regime around *T*_cryostat_ = 110 K, determined by a diffusion coefficient of *D*_0_ = 3.1 Å^2^/s, a compressed exponential behavior (γ > 1.5), and a slight offset in the *Q*^2^ dependence of the relaxation time, which could be related to stress relaxation inside the glassy matrix. Here we investigated temperatures above 110 K, showing the additional oscillatory behavior of *g*_2_. The calculated diffusion coefficient increases to *D*_0_ = 12 and 63 Å^2^/s for 115 and 117.5 K, respectively. Considering γ_2_ ∼ 1 (Figure 3c), the previous finding indicates the formation of a high-density liquid (HDL) at ∼115 K (*T*_sample_ ≈ 120 K), while the stretched exponential behavior γ_2_ < 1 (Figure 3f) at higher temperatures could be related to heterogeneities caused by stress relaxation within the sample. However, the heterodyne signal must be due to a second static component, as discussed above. This might be related to small domains of HDL transforming into LDA, as depicted in Figure 5. LDA seeds can act as a heterodyne static component as its *T*_g_ is higher (136 K) than that of eHDA (115 K). The LDA islands grow and float inside the HDL matrix, which is detected in our small-angle geometry. Alternatively, the appearance of crystalline ice can act as a static component, formed inside the sample or condensed on the surface. However, we consider this scenario to be less likely, as we see the oscillations always appearing simultaneously with the coexistence of the two amorphous states. In addition, the angular dependence as well as the stretched exponential behavior indicates stress-related heterogeneities, potentially due to the preparation process. If the signal originated from condensed ice, it should not exhibit such a strong angular dependence.

**Figure 5:**
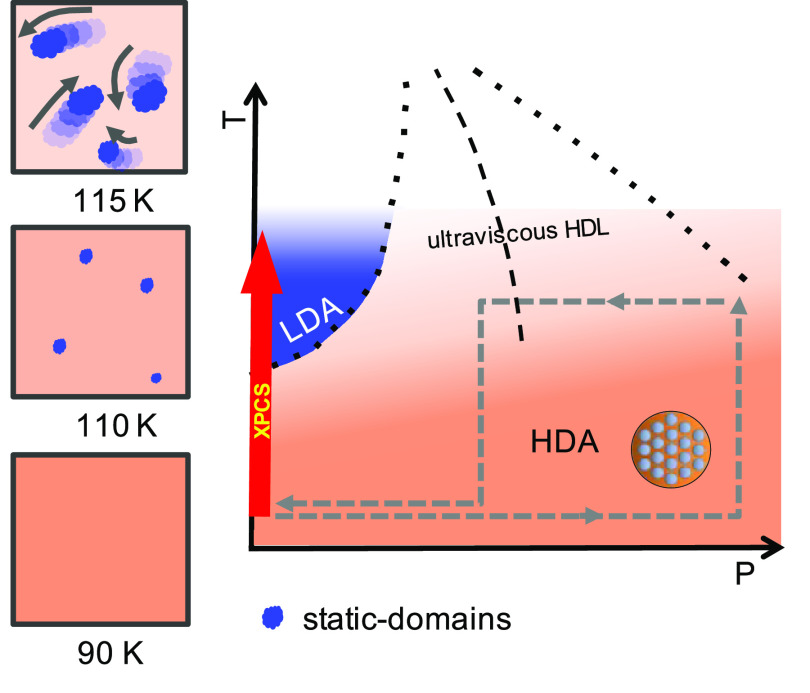
Schematic representation of the structural and dynamic evolutions, including a phase diagram (right) and a preparation pathway (gray dashed). Schematic (left) of sample evolution upon heating at ambient pressure [along red arrow “XPCS” in the phase diagram (right)]. The hypothesis is that HDA first transforms into HDL upon heating to 110 K (*T*_sample_ ≈ 115 K) and further changes to LDA at ∼115 K (*T*_sample_ ≈ 120 K), until it fully transforms at 120 K (*T*_sample_ ≈ 125 K). Oscillatory behavior in the *g*_2_ functions can be explained by static domains that are “swimming” in the HDL matrix.

On the basis of the discussion presented above, we propose a picture for the dynamic and structural evolution of free-standing amorphous ice films (Figure 5). Equilibrated HDA is prepared along the pathway depicted in the phase diagram (Figure 5, right), it remains metastable at ambient pressure and temperatures of ≤90 K. Upon being heated, HDA transforms into a high-density liquid (HDL), which dynamically is slow but diffusive (*D*_0_ = 12 Å^2^/s at 115 K) and is therefore called “ultraviscous”. Within the HDL matrix, small domains then transform into low-density amorphous ice (LDA), as seen in the WAXS signal. Due to the density difference of the two states, expansion takes place when crossing the boundary between HDA and LDA (red arrow in the right ppanel of Figure 5). The appearance of a heterodyne signal (oscillation) must be related to the static component. Possible sources are the slow or almost static LDA domains, condensed crystalline ice, or creep due to expansion. We exclude the possibility of the signal originating from mechanical motion, as this cannot be described by eq 2. The heterodyne signal appears in the *g*_2_ curves at 115 and 117.5 K, hence simultaneously indicating the observation of two coexisting components in WAXS. Once the sample fully transforms into LDA, the heterodyne signal disappears.

In summary, we determined the dynamics during the eHDA–LDA transition by conducting XPCS experiments in transmission geometry (using thin grid samples). A heterodyne signal was surprisingly observed at 115 and 117.5 K (cryostat temperature) when HDL and LDA coexisted within the sample. One of the novelties of our study is the demonstration that such a heterodyne signal can be observed in a homodyne detection scheme in a one-component system and using XPCS in the transmission geometry. Quantitative analysis was performed on this heterodyne signal to extract the intrinsic dynamics of amorphous ice during the HDA–LDA transition. The *Q*^2^ dependence of the extracted relaxation rates and a γ value of ∼1 suggest that a liquid state was formed at relatively high temperature of 115 and 117.5 K (*T*_sample_ values of 120 and 122.5 K, respectively). The higher diffusion coefficient and velocity observed with an increase in temperature further support this conclusion. This leaves us with the scenario depicted in Figure 5 and is consistent with the hypothesized glass transition in HDA at *T* > 110 K and LDA as a static component “swimming” within the liquid matrix. Interestingly, an angular dependence of the heterodyne signal was observed, which might be due to the heterogeneity of the macroscopic morphology inside the free-standing amorphous ice film during the sample preparation process. Overall, the heterodyne signal observed in a homodyne XPCS detection scheme sheds light on the glassy nature of amorphous ices and their phase transitions. This work paves the way for the fundamental studies of other systems where different dynamics and phase transition occur under different conditions.

## Experimental Methods

Details for sample preparation and X-ray measurements can be found in the Supporting Information. In brief, HDA was prepared inside a Cu grid, following our previous protocol,^31^ as depicted in Figure 5. XPCS measurements were performed at beamline P10 and PETRA III at DESY.

## Supporting Information Available

Sample preparation, sample environment, details of X-ray photocorrelation spectroscopy experiments, different models for fitting the *g*_2_ functions, and additional XPCS data sets (PDF)
Transparent Peer Review report available (PDF)

## References

[ref1] Gallo P.; Amann-Winkel K.; Angell C. A.; Anisimov M. A.; Caupin F.; Chakravarty C.; Lascaris E.; Loerting T.; Panagiotopoulos A. Z.; Russo J.; Sellberg J. A.; Stanley H. E.; Tanaka H.; Vega C.; Xu L.; Pettersson L. G. M. Water: A Tale of Two Liquids. Chem. Rev. 2016, 116 (13), 7463–7500. 10.1021/acs.chemrev.5b00750. 27380438 PMC5424717

[ref2] Amann-Winkel K.; Böhmer R.; Fujara F.; Gainaru C.; Geil B.; Loerting T. Colloquium: Water’s Controversial Glass Transitions. Rev. Mod. Phys. 2016, 88 (1), 011002 10.1103/RevModPhys.88.011002.

[ref3] Kim K. H.; Späh A.; Pathak H.; Perakis F.; Mariedahl D.; Amann-Winkel K.; Sellberg J. A.; Lee J. H.; Kim S.; Park J.; Nam K. H.; Katayama T.; Nilsson A. Maxima in the Thermodynamic Response and Correlation Functions of Deeply Supercooled Water. Science 2017, 358, 1589–1593. 10.1126/science.aap8269. 29269472

[ref4] Perakis F.; Amann-Winkel K.; Lehmkühler F.; Sprung M.; Mariedahl D.; Sellberg J. A.; Pathak H.; Späh A.; Cavalca F.; Schlesinger D.; Ricci A.; Jain A.; Massani B.; Aubree F.; Benmore C. J.; Loerting T.; Grübel G.; Pettersson L. G. M.; Nilsson A. Diffusive Dynamics during the High-To-Low Density Transition in Amorphous Ice. Proc. Natl. Acad. Sci. U. S. A. 2017, 114 (31), 8193–8198. 10.1073/pnas.1705303114. 28652327 PMC5547632

[ref5] Kim K. H.; Amann-Winkel K.; Giovambattista N.; Späh A.; Perakis F.; Pathak H.; Ladd Parada M.; Yang C.; Mariedahl D.; Eklund T.; Lane T. J.; You S.; Jeong S.; Weston M.; Lee J. H.; Eom I.; Kim M.; Park J.; Chun S. H.; Poole P. H.; Nilsson A. Experimental Observation of the Liquid-Liquid Transition in Bulk Supercooled Water under Pressure. Science 2020, 370, 978–982. 10.1126/science.abb9385. 33214280

[ref6] Suzuki Y. Direct Observation of Reversible Liquid-Liquid Transition in a Trehalose Aqueous Solution. Proc. Natl. Acad. Sci. U. S. A. 2022, 119, e2113411119 10.1073/pnas.2113411119. 35074875 PMC8812557

[ref7] Debenedetti P. G.; Sciortino F.; Zerze G. H. Second Critical Point in Two Realistic Models of Water. Science 2020, 369, 289–292. 10.1126/science.abb9796. 32675369

[ref8] Nilsson A.; Huang C.; Pettersson L. G. M. Fluctuations in Ambient Water. J. Mol. Liq. 2012, 176, 2–16. 10.1016/j.molliq.2012.06.021.

[ref9] Mishima O.; Stanley H. E. The Relationship between Liquid, Supercooled and Glassy Water. Nature 1998, 396, 329–335. 10.1038/24540.

[ref10] Tulk C. A.; Molaison J. J.; Makhluf A. R.; Manning C. E.; Klug D. D. Absence of Amorphous Forms When Ice Is Compressed at Low Temperature. Nature 2019, 569 (7757), 542–545. 10.1038/s41586-019-1204-5. 31118522

[ref11] Stern J. N.; Seidl-Nigsch M.; Loerting T. Evidence for High-Density Liquid Water between 0.1 and 0.3 GPa near 150 K. Proc. Natl. Acad. Sci. U. S. A. 2019, 116 (19), 9191–9196. 10.1073/pnas.1819832116. 30923121 PMC6511050

[ref12] Rosu-Finsen A.; Davies M. B.; Amon A.; Wu H.; Sella A.; Michaelides A.; Salzmann C. G. Medium-Density Amorphous Ice. Science 2023, 379, 474–478. 10.1126/science.abq2105. 36730416

[ref13] Jain A.; Schulz F.; Lokteva I.; Frenzel L.; Grübel G.; Lehmkühler F. Anisotropic and Heterogeneous Dynamics in an Aging Colloidal Gel. Soft Matter 2020, 16 (11), 2864–2872. 10.1039/C9SM02230A. 32108204

[ref14] Dierker S. B.; Pindak R.; Fleming R. M.; Robinson I. K.; Berman L. X-Ray Photon Correlation Spectroscopy Study of Brownian Motion of Gold Colloids in Glycerol. Phys. Rev. Lett. 1995, 75 (3), 449–452. 10.1103/PhysRevLett.75.449. 10060024

[ref15] Chung B.; Ramakrishnan S.; Bandyopadhyay R.; Liang D.; Zukoski C. F.; Harden J. L.; Leheny R. L. Microscopic Dynamics of Recovery in Sheared Depletion Gels. Phys. Rev. Lett. 2006, 96 (22), 228301 10.1103/PhysRevLett.96.228301. 16803351

[ref16] Lu X.; Mochrie S. G. J.; Narayanan S.; Sandy A. R.; Sprung M. Temperature-Dependent Structural Arrest of Silica Colloids in a Water-Lutidine Binary Mixture. Soft Matter 2010, 6 (24), 6160–6177. 10.1039/c0sm00152j.

[ref17] Sikorski M.; Sandy A. R.; Narayanan S. Depletion-Induced Structure and Dynamics in Bimodal Colloidal Suspensions. Phys. Rev. Lett. 2011, 106 (18), 188301 10.1103/PhysRevLett.106.188301. 21635129

[ref18] Sun Y.; Carini G.; Chollet M.; Decker F. J.; Dunne M.; Fuoss P.; Hruszkewycz S. O.; Lane T. J.; Nakahara K.; Nelson S.; Robert A.; Sato T.; Song S.; Stephenson G. B.; Sutton M.; Van Driel T. B.; Weninger C.; Zhu D. Nonuniform Flow Dynamics Probed by Nanosecond X-Ray Speckle Visibility Spectroscopy. Phys. Rev. Lett. 2021, 127 (5), 058001 10.1103/PhysRevLett.127.058001. 34397240

[ref19] Grübel G.; Zontone F. Correlation Spectroscopy with Coherent X-Rays. J. Alloys Compd. 2004, 362 (1–2), 3–11. 10.1016/S0925-8388(03)00555-3.

[ref20] Falus P.; Borthwick M. A.; Mochrie S. G. J. Fluctuation Dynamics of Block Copolymer Vesicles. Phys. Rev. Lett. 2005, 94 (1), 016105 10.1103/PhysRevLett.94.016105. 15698103

[ref21] Patel A. J.; Narayanan S.; Sandy A.; Mochrie S. G. J.; Garetz B. A.; Watanabe H.; Balsara N. P. Relationship between Structural and Stress Relaxation in a Block-Copolymer Melt. Phys. Rev. Lett. 2006, 96 (25), 257801 10.1103/PhysRevLett.96.257801. 16907344

[ref22] Ruegg M. L.; Patel A. J.; Narayanan S.; Sandy A. R.; Mochrie S. G. J.; Watanabe H.; Balsara N. P. Condensed Exponential Correlation Functions in Multicomponent Polymer Blends Measured by X-Ray Photon Correlation Spectroscopy. Macromolecules 2006, 39 (25), 8822–8831. 10.1021/ma061183y.

[ref23] Brinker K. L.; Mochrie S. G. J.; Burghardt W. R. Equilibrium Dynamics of a Polymer Bicontinuous Microemulsion. Macromolecules 2007, 40 (14), 5150–5160. 10.1021/ma0704820.

[ref24] Patel A. J.; Mochrie S.; Narayanan S.; Sandy A.; Watanabe H.; Balsara N. P. Dynamic Signatures of Microphase Separation in a Block Copolymer Melt Determined by X-Ray Photon Correlation Spectroscopy and Rheology. Macromolecules 2010, 43 (3), 1515–1523. 10.1021/ma902343m.

[ref25] Livet F.; Bley F.; Ehrburger-Dolle F.; Morfin I.; Geissler E.; Sutton M. X-Ray Intensity Fluctuation Spectroscopy by Heterodyne Detection. J. Synchrotron Radiat 2006, 13 (6), 453–458. 10.1107/S0909049506030044. 17057321

[ref26] Berne B. J.; Pecora R. Dynamic Light Scattering with Applications to Chemistry, Biology and Physics; Wiley, 1976.

[ref27] Burghardt W. R.; Sikorski M.; Sandy A. R.; Narayanan S. X-Ray Photon Correlation Spectroscopy during Homogenous Shear Flow. Phys. Rev. E 2012, 85 (2), 021402 10.1103/PhysRevE.85.021402. 22463207

[ref28] Girelli A.; Rahmann H.; Begam N.; Ragulskaya A.; Reiser M.; Chandran S.; Westermeier F.; Sprung M.; Zhang F.; Gutt C.; Schreiber F. Microscopic Dynamics of Liquid-Liquid Phase Separation and Domain Coarsening in a Protein Solution Revealed by X-Ray Photon Correlation Spectroscopy. Phys. Rev. Lett. 2021, 126 (13), 138004 10.1103/PhysRevLett.126.138004. 33861109

[ref29] Sanborn C.; Ludwig K. F.; Rogers M. C.; Sutton M. Direct Measurement of Microstructural Avalanches during the Martensitic Transition of Cobalt Using Coherent X-Ray Scattering. Phys. Rev. Lett. 2011, 107 (1), 015702 10.1103/PhysRevLett.107.015702. 21797551

[ref30] Mariedahl D.; Perakis F.; Späh A.; Pathak H.; Kim K. H.; Benmore C.; Nilsson A.; Amann-Winkel K. X-Ray Studies of the Transformation from High- To Low-Density Amorphous Water. Philosophical Transactions of the Royal Society A 2019, 377 (2146), 20180164 10.1098/rsta.2018.0164. PMC6501918 30982458

[ref31] Karina A.; Eklund T.; Tonauer C. M.; Li H.; Loerting T.; Amann-Winkel K. Infrared Spectroscopy on Equilibrated High-Density Amorphous Ice. J. Phys. Chem. Lett. 2022, 13 (34), 7965–7971. 10.1021/acs.jpclett.2c02074. 35981100 PMC9442797

[ref32] Amann-Winkel K.; Gainaru C.; Handle P. H.; Seidl M.; Nelson H.; Böhmer R.; Loerting T. Water’s Second Glass Transition. Proc. Natl. Acad. Sci. U. S. A. 2013, 110 (44), 17720–17725. 10.1073/pnas.1311718110. 24101518 PMC3816484

[ref33] Winkel K.; Mayer E.; Loerting T. Equilibrated High-Density Amorphous Ice and Its First-Order Transition to the Low-Density Form. J. Phys. Chem. B 2011, 115 (48), 14141–14148. 10.1021/jp203985w. 21793514

[ref34] Ladd-Parada M.; Li H.; Karina A.; Kim K. H.; Perakis F.; Reiser M.; Dallari F.; Striker N.; Sprung M.; Westermeier F.; Grübel G.; Nilsson A.; Lehmkühler F.; Amann-Winkel K. Using Coherent X-Rays to Follow Dynamics in Amorphous Ices. Environmental Science: Atmospheres 2022, 2, 1314–1323. 10.1039/D2EA00052K. 36561555 PMC9648632

[ref35] Nelmes R. J.; Loveday J. S.; Strässle T.; Bull C. L.; Guthrie M.; Hamel G.; Klotz S. Annealed High-Density Amorphous Ice under Pressure. Nat. Phys. 2006, 2 (6), 414–418. 10.1038/nphys313.

[ref36] Lehmkühler F.; Roseker W.; Grübel G. From Femtoseconds to Hours–Measuring Dynamics over 18 Orders of Magnitude with Coherent X-Rays. Applied Sciences 2021, 11, 6179 10.3390/app11136179.

[ref37] Perakis F.; Gutt C. Towards Molecular Movies with X-Ray Photon Correlation Spectroscopy. Phys. Chem. Chem. Phys. 2020, 22 (35), 19443–19453. 10.1039/D0CP03551C. 32870200

[ref38] Madsen A.; Leheny R. L.; Guo H.; Sprung M.; Czakkel O. Beyond Simple Exponential Correlation Functions and Equilibrium Dynamics in X-Ray Photon Correlation Spectroscopy. New J. Phys. 2010, 12, 055001 10.1088/1367-2630/12/5/055001.

[ref39] Williams G.; Watts D. C. Non-Symmetrical Dielectric Relaxation Behaviour Arising from a Simple Empirical Decay Function. Trans. Faraday Soc. 1970, 66, 80–85. 10.1039/tf9706600080.

[ref40] Westermeier F.; Pennicard D.; Hirsemann H.; Wagner U. H.; Rau C.; Graafsma H.; Schall P.; Lettinga M. P.; Struth B. Connecting Structure, Dynamics and Viscosity in Sheared Soft Colloidal Liquids: A Medley of Anisotropic Fluctuations. Soft Matter 2016, 12 (1), 171–180. 10.1039/C5SM01707F. 26451659

[ref41] Ulbrandt J. G.; Rainville M. G.; Wagenbach C.; Narayanan S.; Sandy A. R.; Zhou H.; Ludwig K. F.; Headrick R. L. Direct Measurement of the Propagation Velocity of Defects Using Coherent X-Rays. Nat. Phys. 2016, 12 (8), 794–799. 10.1038/nphys3708.

[ref42] Moulin E.; Nyrkova I. A.; Giuseppone N.; Semenov A. N.; Buhler E. Homodyne Dynamic Light Scattering in Supramolecular Polymer Solutions: Anomalous Oscillations in Intensity Correlation Function. Soft Matter 2020, 16 (12), 2971–2993. 10.1039/C9SM02480H. 32129415

[ref43] Jouault N.; Moulin E.; Giuseppone N.; Buhler E. Light Scattering Strategy for the Investigation of Time-Evolving Heterogeneous Supramolecular Self-Assemblies. Phys. Rev. Lett. 2015, 115 (8), 085501 10.1103/PhysRevLett.115.085501. 26340192

[ref44] Lewis R. M.; Jackson G. L.; Maher M. J.; Kim K.; Lodge T. P.; Mahanthappa M. K.; Narayanan S.; Bates F. S. A New Framework for X-Ray Photon Correlation Spectroscopy Analysis from Polycrystalline Materials. Rev. Sci. Instrum. 2018, 89 (12), 123902 10.1063/1.5051451. 30599637

[ref45] Dallari F.; Martinelli A.; Caporaletti F.; Sprung M.; Grübel G.; Monaco G. Microscopic Pathways for Stress Relaxation in Repulsive Colloidal Glasses. Sci. Adv. 2020, 6 (12), eaaz2982 10.1126/sciadv.aaz2982. 32219168 PMC7083620

[ref46] Giebel B.; Helmbrecht C. Methods to Analyze EVs. Methods Mol. Biol. 2017, 1545, 1–20. 10.1007/978-1-4939-6728-5_1. 27943203

[ref47] Steinrück H. G.; Takacs C. J.; Kim H. K.; MacKanic D. G.; Holladay B.; Cao C.; Narayanan S.; Dufresne E. M.; Chushkin Y.; Ruta B.; Zontone F.; Will J.; Borodin O.; Sinha S. K.; Srinivasan V.; Toney M. F. Concentration and Velocity Profiles in a Polymeric Lithium-Ion Battery Electrolyte. Energy Environ. Sci. 2020, 13 (11), 4312–4321. 10.1039/D0EE02193H.

[ref48] Lhermitte J. R. M.; Rogers M. C.; Manet S.; Sutton M. Velocity Measurement by Coherent X-Ray Heterodyning. Rev. Sci. Instrum. 2017, 88 (1), 015112 10.1063/1.4974099. 28147652

[ref49] Sutton M. A. Review of X-Ray Intensity Fluctuation Spectroscopy. C R Phys. 2008, 9, 657–667. 10.1016/j.crhy.2007.04.008.

[ref50] Busch S.; Jensen T. H.; Chushkin Y.; Fluerasu A. Dynamics in Shear Flow Studied by X-Ray Photon Correlation Spectroscopy. Eur. Phys. J. E 2008, 26 (1–2), 55–62. 10.1140/epje/i2007-10305-2. 18415042

[ref51] Fluerasu A.; Kwasniewski P.; Caronna C.; Destremaut F.; Salmon J. B.; Madsen A. Dynamics and Rheology under Continuous Shear Flow Studied by X-Ray Photon Correlation Spectroscopy. New J. Phys. 2010, 12, 035023 10.1088/1367-2630/12/3/035023.

[ref52] Möller J.; Narayanan T. Velocity Fluctuations in Sedimenting Brownian Particles. Phys. Rev. Lett. 2017, 118, 198001 10.1103/PhysRevLett.118.198001. 28548515

[ref53] Gutt C.; Ghaderi T.; Chamard V.; Madsen A.; Seydel T.; Tolan M.; Sprung M.; Grübel G.; Sinha S. K. Observation of Heterodyne Mixing in Surface X-Ray Photon Correlation Spectroscopy Experiments. Phys. Rev. Lett. 2003, 91 (7), 076104 10.1103/PhysRevLett.91.076104. 12935035

[ref54] Ulbrandt J. G.; Rainville M. G.; Wagenbach C.; Narayanan S.; Sandy A. R.; Zhou H.; Ludwig K. F.; Headrick R. L. Direct Measurement of the Propagation Velocity of Defects Using Coherent X-Rays. Nat. Phys. 2016, 12 (8), 794–799. 10.1038/nphys3708.

[ref55] Ju G.; Xu D.; Highland M. J.; Thompson C.; Zhou H.; Eastman J. A.; Fuoss P. H.; Zapol P.; Kim H.; Stephenson G. B. Coherent X-Ray Spectroscopy Reveals the Persistence of Island Arrangements during Layer-by-Layer Growth. Nat. Phys. 2019, 15 (6), 589–594. 10.1038/s41567-019-0448-1.

[ref56] Moulin E.; Nyrkova I. A.; Giuseppone N.; Semenov A. N.; Buhler E. Homodyne Dynamic Light Scattering in Supramolecular Polymer Solutions: Anomalous Oscillations in Intensity Correlation Function. Soft Matter 2020, 16 (12), 2971–2993. 10.1039/C9SM02480H. 32129415

[ref57] Jouault N.; Moulin E.; Giuseppone N.; Buhler E. Light Scattering Strategy for the Investigation of Time-Evolving Heterogeneous Supramolecular Self-Assemblies. Phys. Rev. Lett. 2015, 115 (8), 085501 10.1103/PhysRevLett.115.085501. 26340192

