# Supplementary material for: Intrinsic Dynamics of Amorphous Ice Revealed by a Heterodyne Signal in X-ray Photon Correlation Spectroscopy Experiments
Source: J Phys Chem Lett. 2023 Dec 1;14(49):10999–1007. doi: 10.1021/acs.jpclett.3c02470 (PMC10726389; doi:10.1021/acs.jpclett.3c02470)
Supplement: Supplementary file 1 — jz3c02470_si_001.pdf [file jz3c02470_si_001.pdf]

Supporting information for
Intrinsic Dynamics of Amorphous Ice Revealed by a Heterodyne Signal in X-Ray Photon Correlation Spectroscopy Experiments

Hailong Li[1,2], Marjorie Ladd-Parada[3,4], Aigerim Karina[3], Francesco Dallari[5,6], Mario Reiser[3], Fivos Perakis[3], Nele N. Striker[5], Michael Sprung[5], Fabian Westermeier[5], Gerhard Grübel[5,7,8], Werner Steffen[1], Felix Lehmkühler[5,7], Katrin Amann-Winkel[1,3,9]*

[1]Max-Planck-Institute for Polymer Research, Ackermannweg 10, 55128 Mainz, Germany
[2]State Key Laboratory of Fine Chemicals, School of Chemical Engineering, Dalian University of Technology, Dalian 116024, China
[3]Department of Physics, AlbaNova University Centre, Stockholm University, 10691 Stockholm, Sweden
[4]Department of Chemistry, KTH Royal Institute of Technology, Roslagstullsbacken 21, 11421 Stockholm, Sweden
[5]Deutsches Elektronen-Synchrotron DESY, Notkestr. 85, 22607 Hamburg, Germany
[6]University of Padova, Department of Physics and Astronomy "Galileo Galilei", 35131 Padova, Italy
[7]Hamburg Centre for Ultrafast Imaging, Luruper Chaussee 149, 22761 Hamburg, Germany
[8]European X-ray Free-Electron Laser, Holzkoppel 4, 22869 Schenefeld
[9]Institute of Physics, Johannes Gutenberg University Mainz, Staudingerweg 7, 55128 Mainz, Germany
*Corresponding author: Katrin Amann-Winkel, Email: amannk@mpip-mainz.mpg.de

Sample preparation

60-80 μm thick free-standing amorphous ice films were prepared inside Cu grid holes by using a piston cylinder setup (ZWICK Z100 TN) and stored in liquid nitrogen until use. The preparation pathway (grey dashed arrow) is sketched in the phase diagram in Fig. 5: a Cu-disk with several large holes (diameter of single hole = 1.5 mm) was filled with crystalline ice from ultrapure deionized water. HDA was formed through compression at 100 K. After annealing at 1.1 GPa to 160 K, the sample was decompressed to 0.08 GPa at around 140 K, allowing the transformation into equilibrated HDA (eHDA). A detailed sample preparation method can be found in our previous work.[1–3] Previous X-ray[2] and infrared[1] measurements confirm that eHDA formed inside the grid holes using this procedure. However, the term eHDA here only refers to the preparation pathway [2, 3], and not to a fully expanded eHDA as demonstrated by Nelmes et al.[4]. How sample preparation and friction can lead to slight shifts in the first diffraction maximum was discussed in literature.[5] Here, in addition the sample is free standing in vacuum, which might result in a shift in thermal stability. Furthermore, the sample temperature cannot be measured directly and is estimated to be + 5 K compared to the cryostat temperature.[3]

X-ray photon correlation spectroscopy experiments

XPCS in ultra-small-angle (USAXS) geometry was performed at beamline P10, PETRA III at DESY to study the dynamics of free-standing amorphous ice films during the eHDA-LDA transition, while the corresponding structural change was monitored simultaneously by WAXS. An unfocused X-ray beam with size of 100 μm × 100 μm and a photon energy of 8.4 keV was used. To avoid any beam induced damage to the sample, we used 16 absorbers [3], which refers to the number of Si foils (each foil with a thickness of 25 μm) and results in a final X-ray flux of 6.85×10^8 photons/s at an area of 100 μm x100 μm. The XPCS patterns were recorded with an Eiger X4M detector, located 21.2 m downstream from the sample. An Eiger 500K detector was located 147 mm from the sample to record the WAXS patterns. The mapping from detector pixels (Fig. 1a) to momentum transfer Qs (Fig. 1b) is calibrated by measuring LaB6 as a reference powder sample. The experimental geometry (spatial relationship between beam, sample, and detector) is obtained from a least-squares fit to selected points placed on the Debye-Scherrer rings, as they appear on the detector, using the calibration utilities included in the PyFAI Python library.[6]

Sample environment

The XPCS measurements were performed using a JANIS liquid nitrogen cryostat (VPF-100). The ice sample was mounted on a customized sample holder within a vacuum chamber (10^{-5} mbar), which was directly connected to the X-ray path to avoid any additional scattering from window material. This is, the ice film is free standing in vacuum, without protecting windows. The sample temperature was measured by using a Si-diode mounted at the bottom of the cold-finger. All temperatures stated in this work are the measured cryostat-temperatures ($T_{cryostat}$). We expect a small offset between the given cryostat-temperature and the sample temperature itself, due to varying thermal contact when loading the sample under cryogenic conditions. The sample temperature (T_{sample}) was estimated to be up to 5 K higher than the given cryostat-temperature.[3] We cannot exclude small temperature gradients inside the sample, due to different thermal contact of ice inside each sample compartment of the grid.

Small-angle-X-ray scattering: integrated SAXS intensities

We simultaneously recorded the scattering intensity in wide and small-angle geometry using two detectors (see X-ray photon correlation spectroscopy experiments section). Here, the scattering intensity recorded in SAXS using the Eiger X4M after azimuthal integration is plotted as I(Q) for different temperatures in Fig. S1.

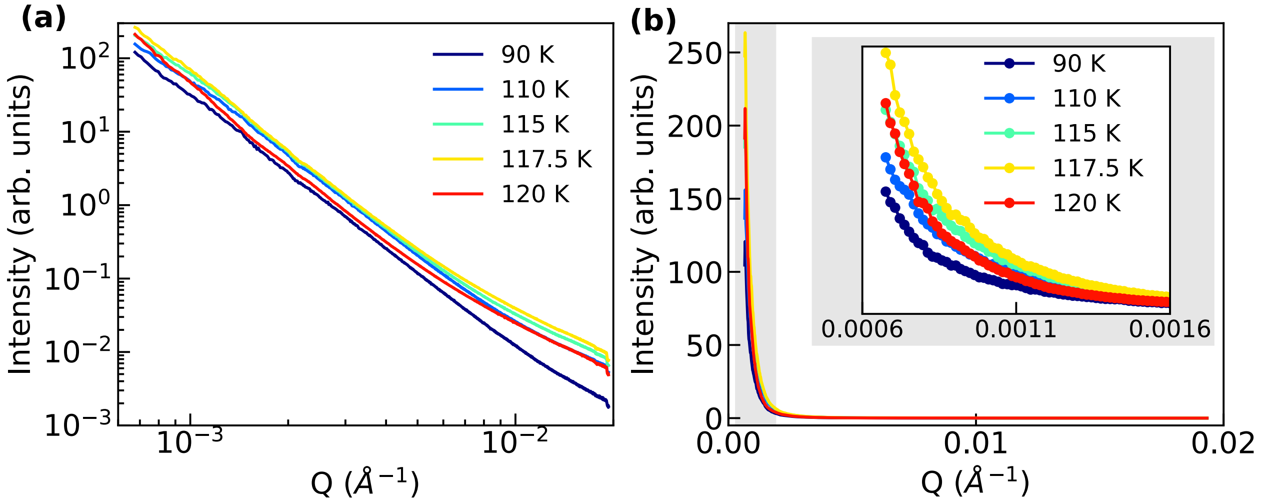

Fig. S1. I(Q) at different temperatures in log-log scale (a) and linear-linear scale (b). Inset in (b) shows a zoom into the low Q-values.

Fast decay process

As shown by the intensity autocorrelation functions in Fig. 1c of the main text, the speckle contrast slightly decreases with increasing Q values for short-delay time for all temperatures, which can be assigned to a fast decay process. Such an effect was also observed in other studies, as e.g. colloidal gels[7]. Using instead an aerogel as reference, no contrast drop is observed (Fig. S2a).

Following the examples [7] where such a drop is observed, the size of the localized motion r_{loc} can be extracted from the equations below:[7]

$$\mathrm{F}(Q,\infty) = \sqrt{b/b_0} = \sqrt{{f_0}^2 \exp(-2) Q^2 \langle {r_{loc}}^2 \rangle} \quad \text{(S1)}$$

$$\log(b/b_0) = 2\log(f_0) - 2Q^2 \langle {r_{loc}}^2 \rangle \quad \text{(S2)}$$

where b and b_0 are the speckle contrast taken from the shorter-delay time plateau value of the g_2 functions of the ice and aerogel samples, respectively.

Figure S2b presents the negative logarithm of the ratio b/b_0 *vs.* Q^2 for the faster decay process at different temperatures. A length scale of around 45 Å was determined for such a localized process at all temperatures.

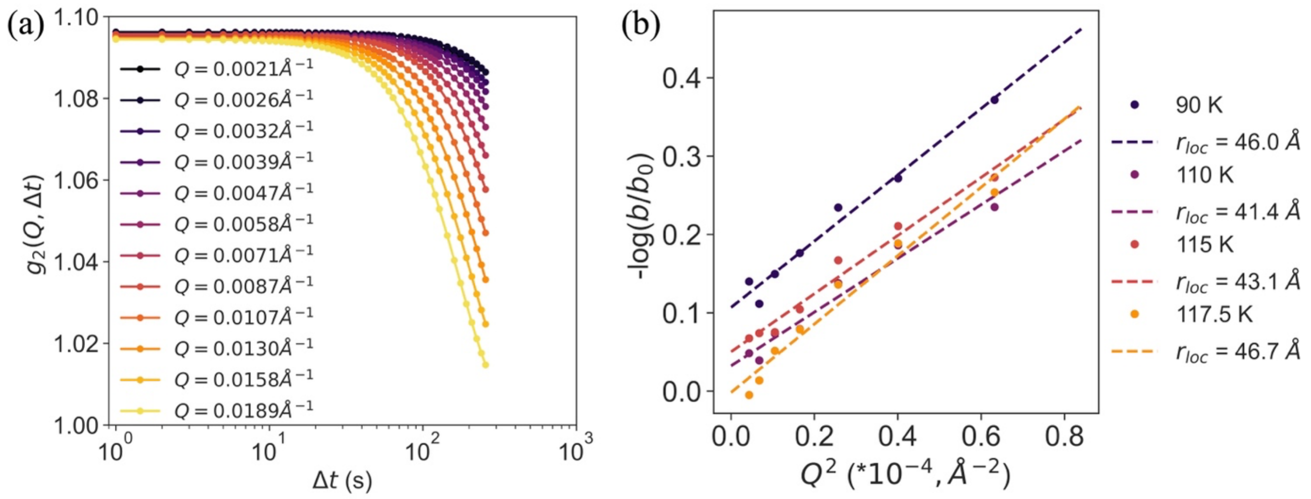

Fig. S2. a): Aerogel reference sample, here no contrast drop is visible. **b)**: Evaluation of contrast drop in HDA samples. Related to eHDA-data of figure 1 in main manuscript. Negative logarithm of the ratio b/b_0 *vs.* Q^2 for the faster decay process at different temperatures. Dashed lines are linear fits using Eq. S2.

Fitting g_2 functions with different models

A sum of Kohlrausch-Williams-Watts (KWW) functions[8] as shown in Eq. S3 is applied for fitting the g_2 functions. Six representative datasets and the corresponding fitting results are plotted in Fig. S3. It is obvious that the fitting is not satisfactory and Eq. S3 is thus not suitable for these datasets.

$$g_2(Q,\Delta t) = 1 + A\exp\left[-1\left(2\frac{\Delta t}{\tau_1}\right)^{\gamma_1}\right] + B\exp\left[-1\left(2\frac{\Delta t}{\tau_2}\right)^{\gamma_2}\right] \qquad \text{(S3)}$$

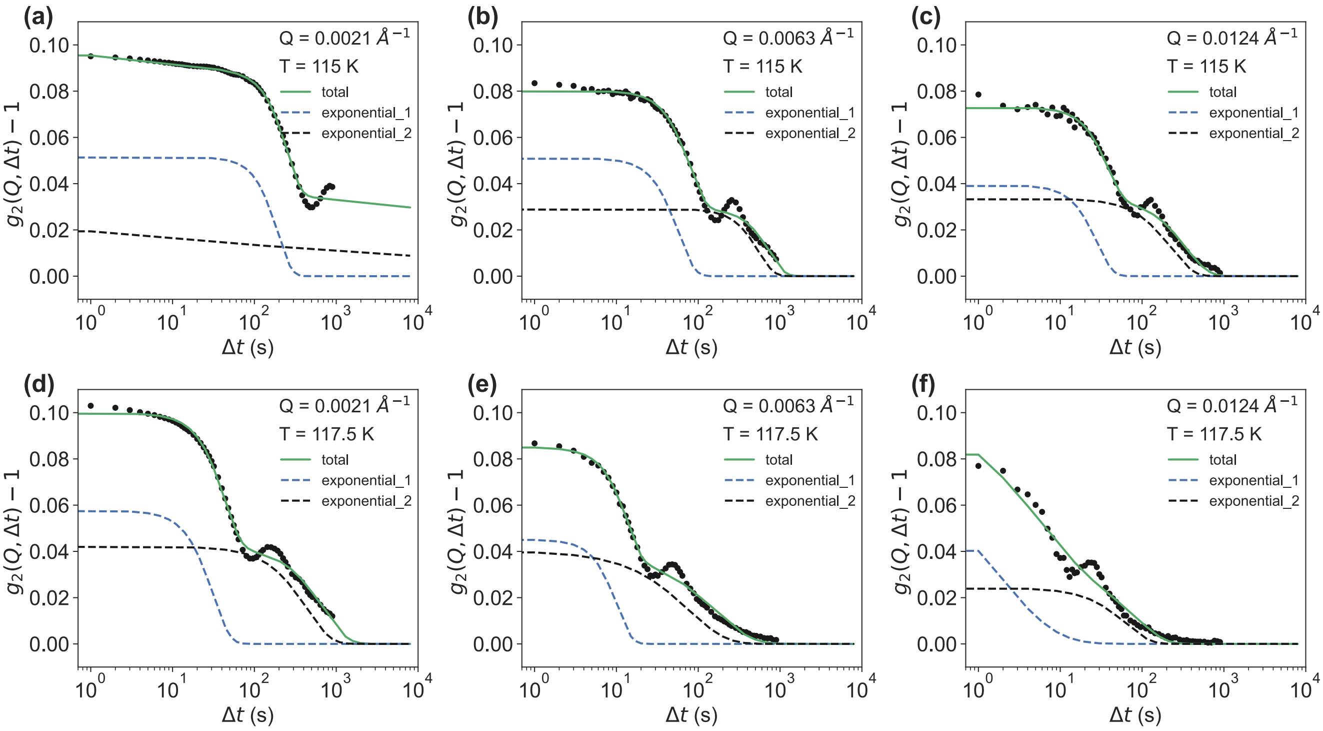

Fig. S3. Representatives of experimental data (black dots) showing oscillations obtained at 115 K (a-c) and 117.5 K (d-f) for 3 different Q-values per temperature. The data are fitted with two exponential functions (Eq. S3): the green solid, black dashed, and blue dashed lines indicate the total, first- and second exponential component of Eq. S3, respectively.

In a second try, a homogeneous shear flow model,[9] neglecting transit effects, is selected to fit the g_2 functions (Eq. S4). The same representative datasets as displayed in Fig. S3 and the corresponding fitting results are plotted in Fig. S4. Also, this model is not able to adequately describe the g_2 functions.

$$g_2(Q,\Delta t) = 1 + \beta \exp[-2Dq^2\Delta t] \frac{\sin^2(\Gamma\Delta t)}{(\Gamma\Delta t)^2} \quad \text{(S4)}$$

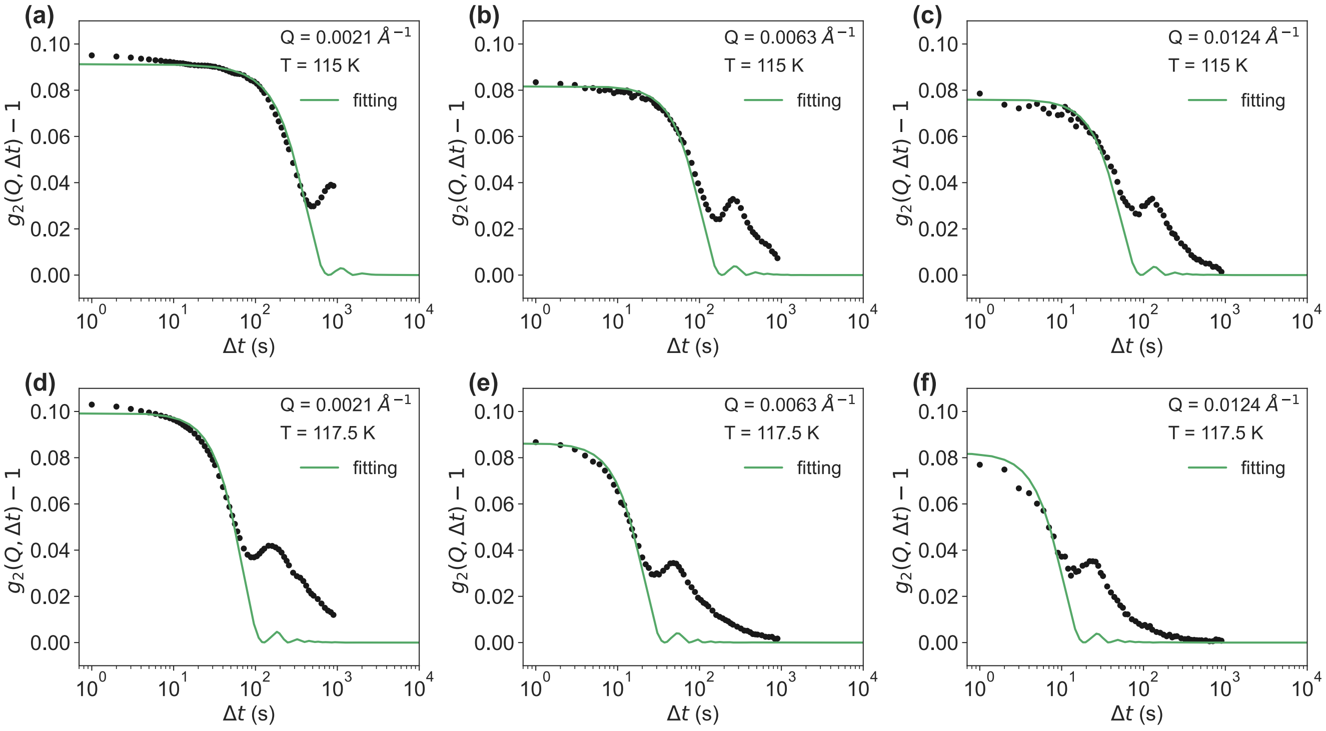

Fig. S4. Representatives of experimental data (black dots) showing oscillations obtained at 115 K (a-c) and 117.5 K (d-f) for 3 Q-values per temperature. The data are fitted with a shear flow model (Eq. S4): the green solid lines indicate the final fitting results.

A similar shear flow model,[10] but with a modified formula (Eq. S5), is also checked. Six representative datasets and the corresponding fitting results are plotted in Fig. S5. The fitting results are getting closer to the data, but a strong discrepancy remains. Therefore, we conclude that a shear flow model is not suitable to describe our system.

$$g_2(Q, \Delta t) = 1 + \beta \left((1 - b)\, exp[-2\Gamma \Delta t] + b \left(\frac{sin(\Gamma_s \Delta t)}{\Gamma_s \Delta t} \right)^2 \right) \qquad \text{(S5)}$$

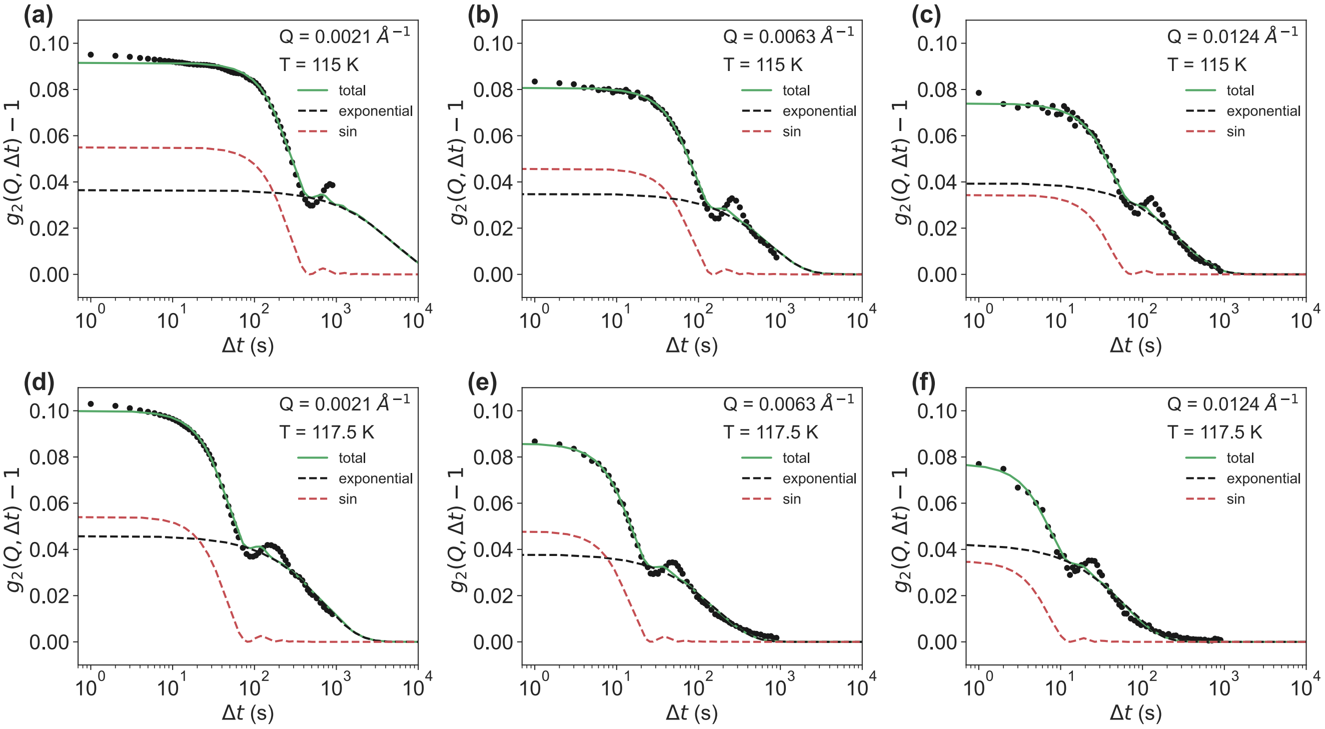

Fig. S5. Representatives of experimental data (black dots) showing oscillations obtained at 115 K (a-c) and 117.5 K (d-f) for 3 different Q-values per temperature. The data are fitted with a modified shear flow model (Eq. S5): the green solid, black dashed, and red dashed lines indicate the total, the exponential component, and the oscillatory component of the model, respectively.

We therefor used a modified model to describe our data, as displayed by Eq. 2 in the main text, the fitting results at 6 different Q-values are displayed in Fig. S6 and S7 for datasets taken at a temperature of 115 K and 117.5 K, respectively. It is observed that this fitting model displays the oscillations very well at different Q-values and different temperatures (Fig. 3a, 3d, S6, and S7). The characteristic times, τ_1 and τ_2, obtained from the fitting of the first and second exponential components at different Q-values at 115 and 117.5 K are summarized in Fig. S14 and 3, respectively.

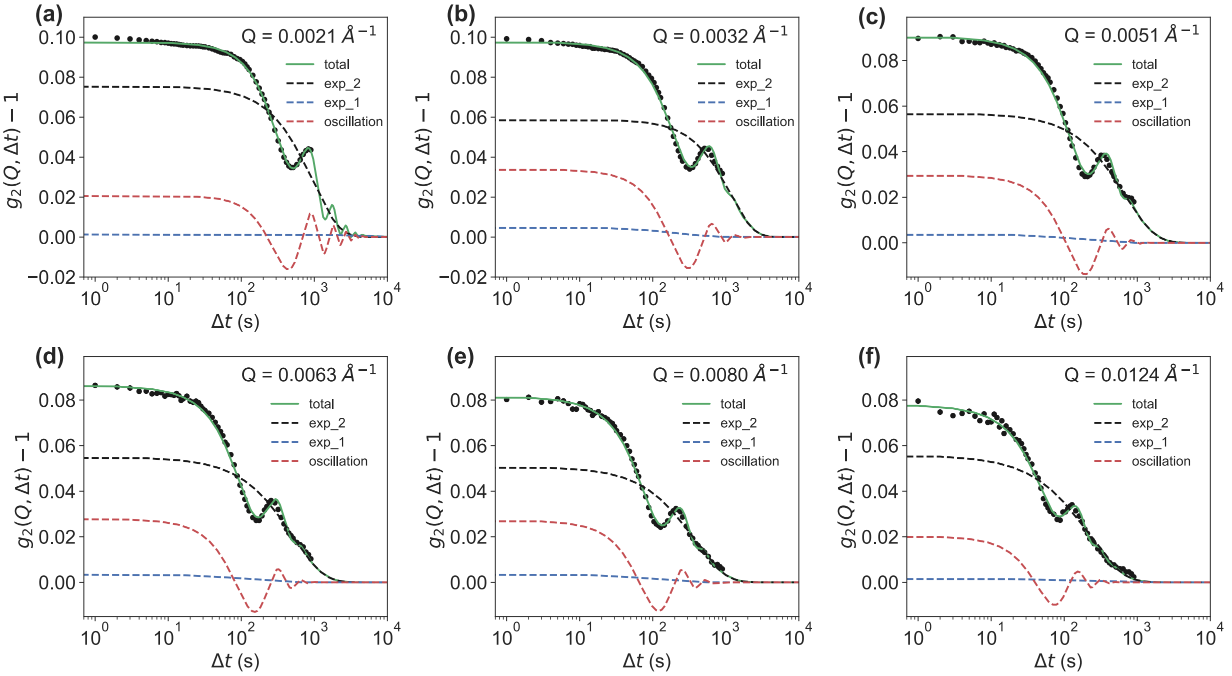

Fig. S6. Quantitative analysis of the g_2 curves. Representatives of experimental data (black dots) showing oscillations obtained at 6 different Q for 115 K. The data are fitted with Eq. 2 in the main text: the green solid, black dashed, blue dashed and red dashed lines indicate the total, first- and second exponential component, and the oscillatory component of the model, respectively.

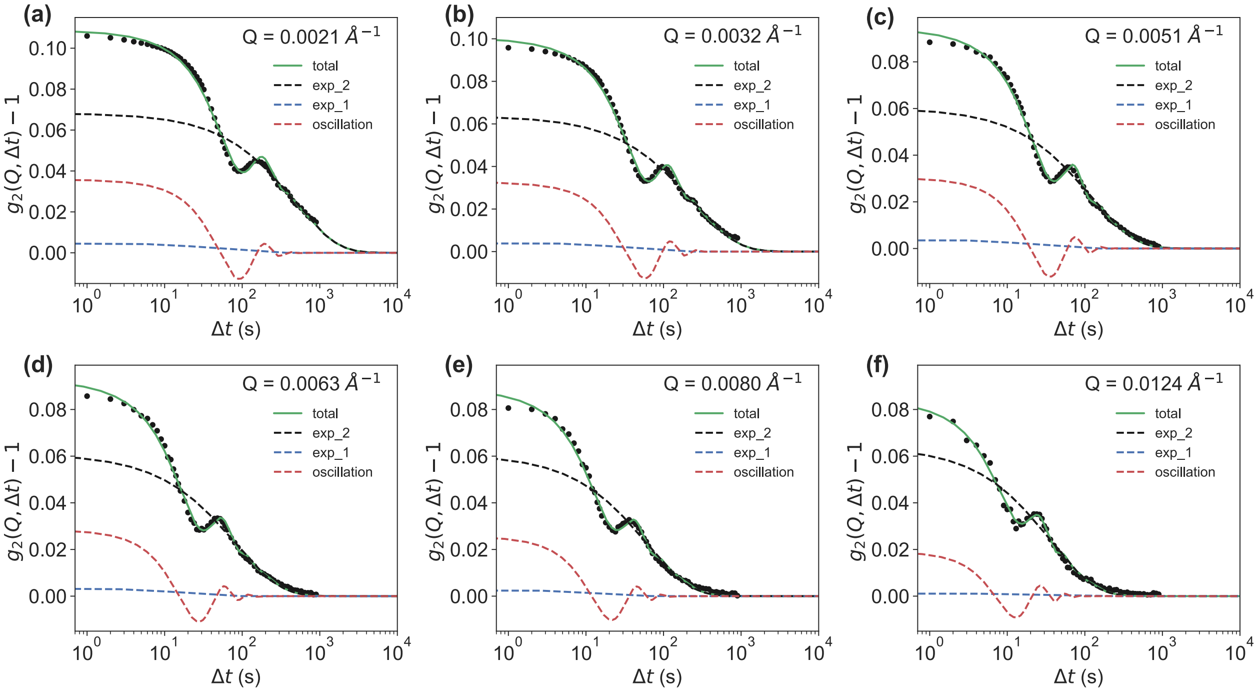

Fig. S7. Quantitative analysis of the g_2 curves. Representatives of experimental data (black dots) showing oscillations obtained at 6 different Q for 117.5 K. The data are fitted with Eq. 2 in the main text: the green solid, black dashed, blue dashed and red dashed lines indicate the total, first- and second exponential component, and the oscillatory component of the model, respectively.

The intensity factors, I_1^2/I_{total}^2 and I_2^2/I_{total}^2, obtained from the fitting of the first and second exponential components (Eq. 2 in main text) at different Q-values at 115 and 117.5 K are summarized in Fig. S8a and S8c, respectively. It is observed that the I_2^2/I_{total}^2 values are one order of magnitude higher than I_1^2/I_{total}^2 values in the whole Q range, indicating that the second exponential component dominates. This fitting result supports us to mainly focus on the second exponential component in this work. The ratios of I_1/I_2 at different Q at 115 and 117.5 K were calculated based on the results presented in Fig. S8a and S8c, and are shown in Fig. S8b and S8d, respectively. From this result we can exclude that the oscillatory signal originates from surface roughness.[11]

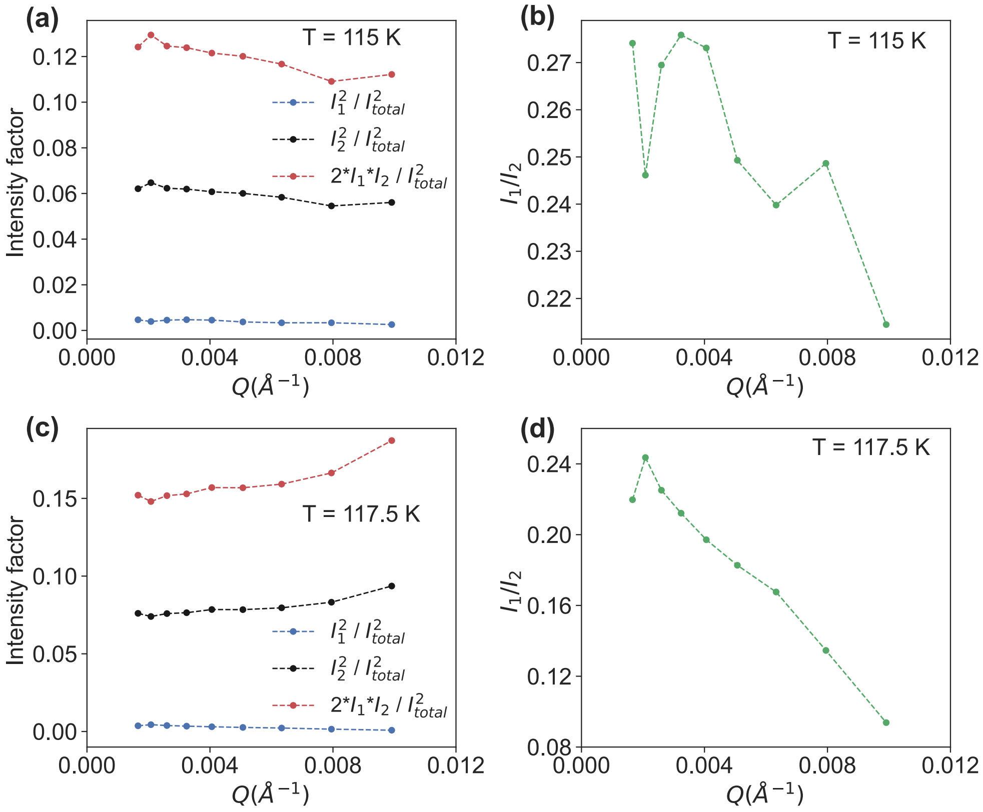

Fig. S8. Quantitative analysis of the g_2 curves. The intensity factors (a) and (c) obtained from the fitting results as listed in Fig. 3 in the main text at different Q at 115 and 117.5 K, respectively. (b) and (d) The ratio of I_1/I_2 at different Q at 115 and 117.5 K, respectively.

Angular dependence of sample A

2D SAXS patterns and g_2 functions at different azimuthal angles ϕ at Q = 0.0041 Å^{-1} for 5 different temperatures for the same sample (sample A) are presented in Fig. S9. The oscillatory behavior can be observed at 115, 117.5 and 120 K, which are above the glass transition onset temperature of equilibrated HDA T_g (eHDA) = 115 K.

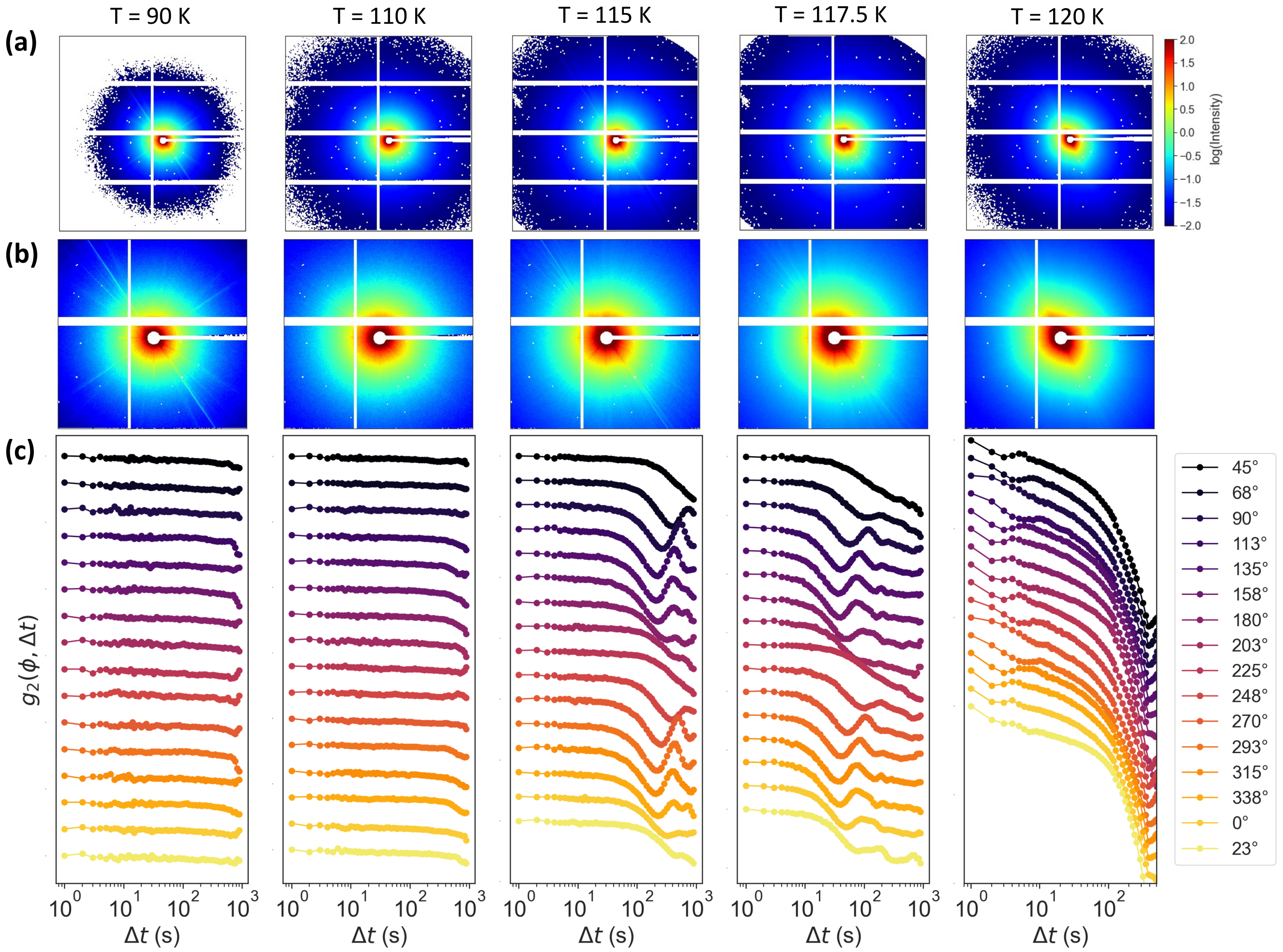

Fig. S9. Angular dependent dynamics of sample A. (a) 2D small angle X-ray scattering patterns. (b) Zoom into the low Q region of the 2D SAXS patterns. (c) Intensity autocorrelation functions $g_2(Q, \Delta t)$ calculated for different angles at Q = 0.0041 Å^{-1}. The g_2 curves are shifted vertically for clarity. Note: the streaks which are visible in Fig. S9b were masked for the calculation of the $g_2(Q, \Delta t)$ functions.

Angular dependence of sample B

Identical XPCS experiments were conducted on another sample B at position 1 (namely B1). The 2D SAXS patterns and g_2 functions at different azimuthal angles ϕ and Q-values for 6 different temperatures are presented in Fig. S10. The oscillatory behavior can also be observed in the long delay time at 117.5 and 120 K (Fig. S10c and S10d). The corresponding SAXS and WAXS curves are presented in Fig. S11. The bimodal scattering intensity from eHDA and LDA in WAXS measured at 120 K implies that this sample spot is rather stable compared to sample A.

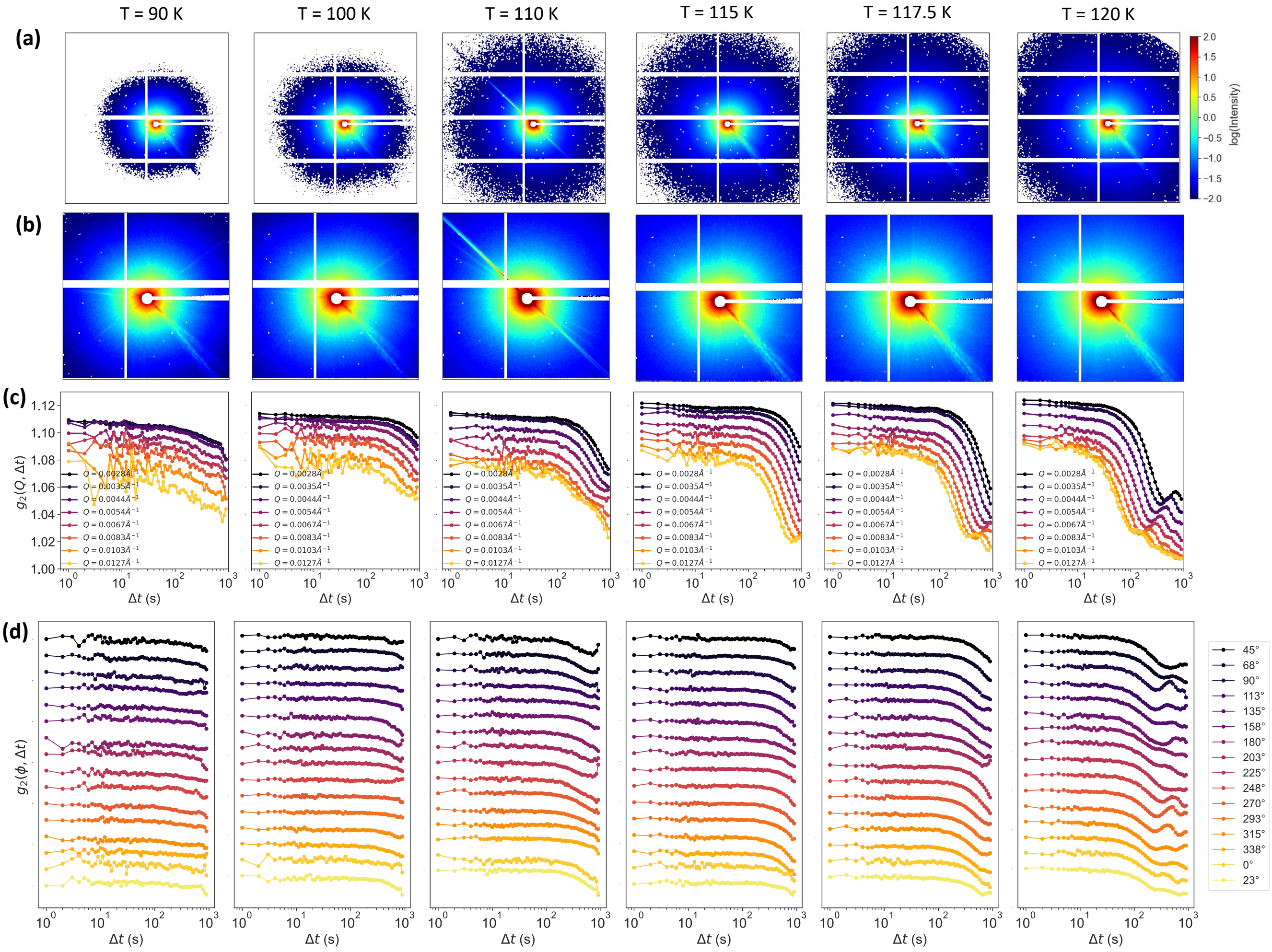

Fig. S10. Angular dependent dynamics of sample B at position 1 (namely B1). (a) 2D small angle X-ray scattering patterns. (b) Zoom into the low Q region of the 2D SAXS patterns. (c) Averaged intensity autocorrelation functions $g_2(Q, \Delta t)$ calculated at different Q for the corresponding temperatures. (d) $g_2(Q, \Delta t)$ calculated for different angles at $Q = 0.0044$ Å^{-1}. The g_2 curves are shifted vertically for clarity. Note: the streaks in Fig. S10b were masked for the calculation of the $g_2(Q, \Delta t)$ functions.

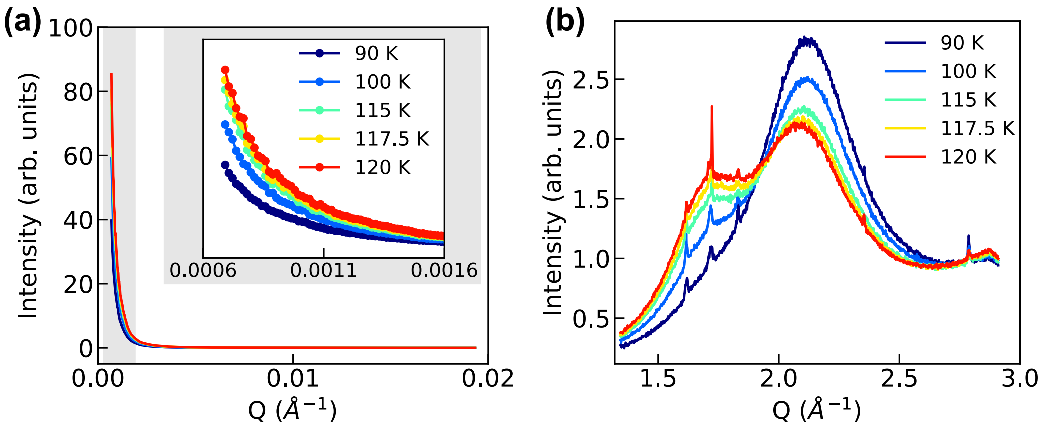

Fig. S11. I(Q) of sample B at position 1 (namely B1). (a) SAXS I(Q) recorded at different temperatures. The inset shows a zoom into the low Q region. (b) WAXS I(Q) recorded at different temperatures.

Angular dependence of sample B

XPCS measurements were also performed on sample B at another position 2 (namely B2) at 115, 117.5 and 120 K. The 2D SAXS patterns and g_2 functions at different azimuthal angles ϕ and Q-values are presented in Fig. S12. The corresponding SAXS and WAXS curves are presented in Fig. S13. Strong oscillations were observed for this sample spot, where crystalline ice signal was observed on WAXS curves. In particular the WAXS curve of T = 120 K (Fig. S13b, red curve) indicates presence of hexagonal ice, rather than cubic ice, hence the crystalline ices most likely formed by condensation on the surface. This measurement was done at a rather stable sample spot, which to a large degree is still consisting of eHDA at 120 K. Nevertheless, we see strong oscillations in the correlation function, once a shoulder at the LDA position appears.

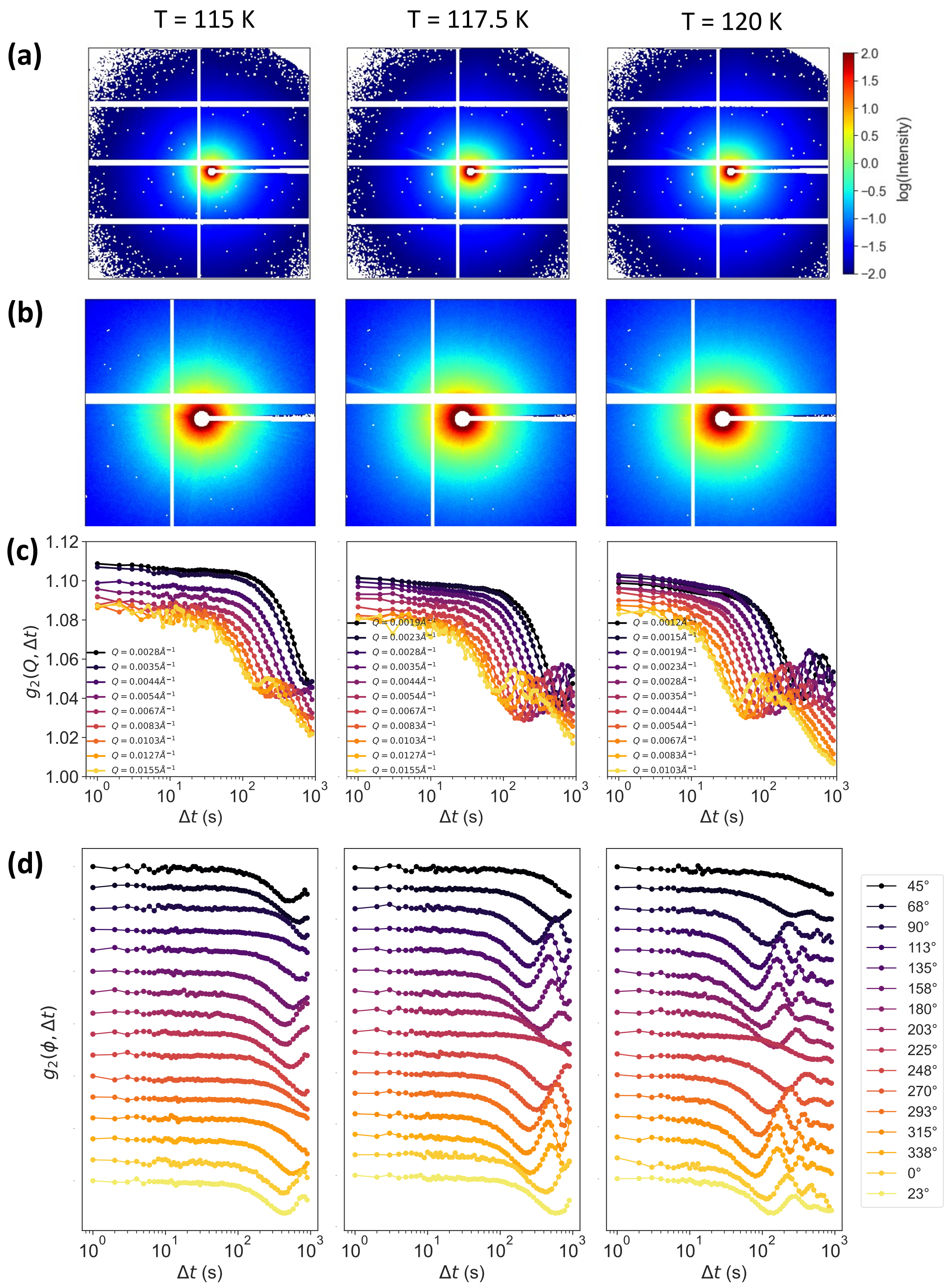

Fig. S12. Angular dependent dynamics of sample B at another position 2 (namely B2). (a) 2D small angle X-ray scattering patterns. (b) Zoom into the low Q region of the 2D SAXS patterns. (c) Averaged intensity autocorrelation functions $g_2(Q, \Delta t)$ calculated at different Q for the corresponding temperatures. (d) $g_2(Q, \Delta t)$ calculated for different angles at $Q = 0.0044$ Å^{-1}. The g_2 curves are shifted vertically for clarity. Note: the streaks in Fig. S12b were masked for the calculation of the $g_2(Q, \Delta t)$ functions.

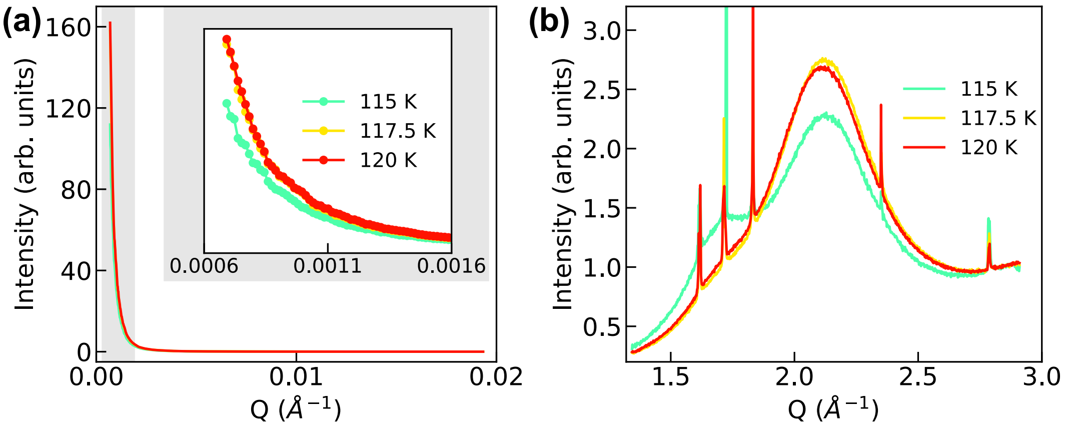

Fig. S13. I(Q) of sample B at position 2 (namely B2). (a) SAXS I(Q) recorded at different temperatures. The inset shows a zoom into the low Q region. (b) WAXS I(Q) recorded at different temperatures.

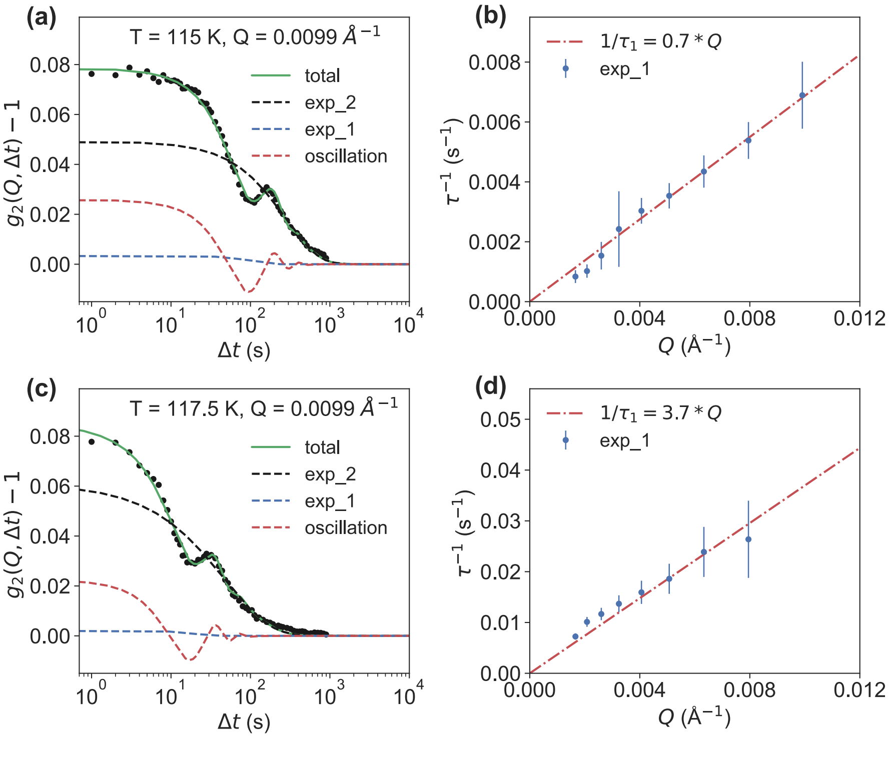

Fig. S14. Same data as shown in Fig. 3 in the main text, but additionally displaying the Q-dependence of τ_1. (a, c) Representatives of experimental data (black dots) showing oscillations obtained at Q = 0.0099 $Å^{-1}$ for 115 and 117.5 K, respectively. The data are fitted with Eq. 2: the green solid, black dashed, blue dashed and red dashed lines indicate the total, first- and second exponential component, and the oscillatory component of the model, respectively. (b, d) The characteristic time, τ_1, obtained from fitting of the first exponential component at different Q values at 115 and 117.5 K, respectively.

References

(1) Karina, A.; Eklund, T.; Tonauer, C. M.; Li, H.; Loerting, T.; Amann-Winkel, K. Infrared Spectroscopy on Equilibrated High-Density Amorphous Ice. *J Phys Chem Lett* **2022**, *13* (34), 7965–7971.

(2) Kim, K. H.; Amann-Winkel, K.; Giovambattista, N.; Späh, A.; Perakis, F.; Pathak, H.; Ladd Parada, M.; Yang, C.; Mariedahl, D.; Eklund, T.; Lane, T. J.; You, S.; Jeong, S.; Weston, M.; Lee, J. H.; Eom, I.; Kim, M.; Park, J.; Chun, S. H.; Poole, P. H.; Nilsson, A. Experimental Observation of the Liquid-Liquid Transition in Bulk Supercooled Water under Pressure. *Science* **2020**, *370*, 978–982.

(3) Ladd-Parada, M.; Li, H.; Karina, A.; Kim, K. H.; Perakis, F.; Reiser, M.; Dallari, F.; Striker, N.; Sprung, M.; Westermeier, F.; Grübel, G.; Nilsson, A.; Lehmkühler, F.; Amann-Winkel, K. Using Coherent X-Rays to Follow Dynamics in Amorphous Ices. *Environmental Science: Atmospheres* **2022**, *2*, 1314–1323.

(4) Nelmes, R. J.; Loveday, J. S.; Strässle, T.; Bull, C. L.; Guthrie, M.; Hamel, G.; Klotz, S. Annealed High-Density Amorphous Ice under Pressure. *Nat Phys* **2006**, *2* (6), 414–418.

(5) Mariedahl, D.; Perakis, F.; Späh, A.; Pathak, H.; Kim, K. H.; Benmore, C.; Nilsson, A.; Amann-Winkel, K. X-Ray Studies of the Transformation from High- To Low-Density Amorphous Water. *Philosophical Transactions of the Royal Society A: Mathematical, Physical and Engineering Sciences* **2019**, *377* (2146).

(6) Ashiotis, G.; Deschildre, A.; Nawaz, Z.; Wright, J. P.; Karkoulis, D.; Picca, F. E.; Kieffer, J. The Fast Azimuthal Integration Python Library: PyFAI. *J Appl Crystallogr* **2015**, *48*, 510–519.

(7) Jain, A.; Schulz, F.; Lokteva, I.; Frenzel, L.; Grübel, G.; Lehmkühler, F. Anisotropic and Heterogeneous Dynamics in an Aging Colloidal Gel. *Soft Matter* **2020**, *16* (11), 2864–2872. https://doi.org/10.1039/c9sm02230a.

(8) Williams, G.; Watts, D. C. Non-Symmetrical Dielectric Relaxation Behaviour Arising from a Simple Empirical Decay Function. *Transactions of the Faraday Society* **1970**, *66*, 80–85.

(9) Burghardt, W. R.; Sikorski, M.; Sandy, A. R.; Narayanan, S. X-Ray Photon Correlation Spectroscopy during Homogenous Shear Flow. *Phys Rev E* **2012**, *85* (2), 021402.

(10) Westermeier, F.; Pennicard, D.; Hirsemann, H.; Wagner, U. H.; Rau, C.; Graafsma, H.; Schall, P.; Lettinga, M. P.; Struth, B. Connecting Structure, Dynamics and Viscosity in Sheared Soft Colloidal Liquids: A Medley of Anisotropic Fluctuations. *Soft Matter* **2015**, *12* (1), 171–180.

(11) Dallari, F.; Martinelli, A.; Caporaletti, F.; Sprung, M.; Baldi, G. I.; Monaco, G. Stochastic Atomic Acceleration during the X-Ray-Induced Fluidization of a Silica Glass. *Proc Natl Acad Sci U S A* **2023**, *120* (2), e2213182120.
