# Supplementary material for: Intrinsic Dynamics of Amorphous Ice Revealed by a Heterodyne Signal in X-ray Photon Correlation Spectroscopy Experiments
Source: J Phys Chem Lett. 2023 Dec 1;14(49):10999–1007. doi: 10.1021/acs.jpclett.3c02470 (PMC10726389; doi:10.1021/acs.jpclett.3c02470)
Supplement: Supplementary file 2 — jz3c02470_si_002.pdf [file jz3c02470_si_002.pdf]

jz-2023-024704.R1

Name: Peer Review Information for "Intrinsic Dynamics of Amorphous Ice Revealed by a Heterodyne Signal in X-Ray Photon Correlation Spectroscopy Experiments"

First Round of Reviewer Comments

Reviewer: 1

Comments to the Author

Li et al. presented an observation of novel dynamics in amorphous ice supported by heterodyne XPCS. The system itself has been studied by the same team (Perakis et al., 114 (31) 8193 2017, Ref. 4), but the observation of heterodyne is quite intriguing. I would recommend publication of the manuscript if the following concerns are addressed:

1. In the XPCS heterodyne analysis, as documented in Ref. 40 (Ulbrandt et al.) and Lewis et al., Rev. Sci. Instrum. 89, 123902 (2018), Q is treated as a vector instead of a scalar. In the scenario where the velocity is of the same amplitude but in opposite directions (e.g., when the sample is under rheological shear and the x-ray beam shines through the middle of the rheometer), such motion may lead to the oscillation of velocity, as shown in Fig. 4d of the manuscript. This might provide additional information to the physical picture of the dynamics. Have the authors considered fitting the phi-dependent g2 with Eq. 8 in Lewis et al.?

2. Have the authors provided background scattering intensity? Static sample holders or windows could result in heterodyning if the intensity is comparable. Such a scenario is unlikely given that the sample is free-standing and the heterodyning is temperature-dependent (Fig. 1-2), however it would be ideal to clarify this as a sanity check.

3. The authors attributed the change of contrast in Fig. 1C to fast dynamics. However, it appears more of an artifact in XPCS analysis as the g2's at different Q are flat at fast time scales and simply offsetted instead of showing a double-stage and impartial decay. Have the authors considered the possible effect from the intensity variation in the Q-average regions? Some examples include Section 6 and 7 of SI in Xi et al., Soft Matter, 2023, 19, 233 and Fig. S6 in Sheyfer et al., Phys. Rev. Lett. 125 125504 (2020).

4. Fig. 1a shows the 2D detector images with the pixel number as the axis, which does not directly correspond to the Q values and is a mismatch to Fig. 1b. Also, the Q region at which Fig. 1c was calculated did not appear in Fig. 1a and 1b. Is there a particular reason for not showing the 1D and 2D SAXS in those regions?

5. The offset of g2 in Fig. 4b is not specified. Also, some g2s have larger overall decay than the others, which is likely caused by the same reason in Comment 3. This may have also caused the crossover between 225 and 203 degrees which looks quite confusing.

Reviewer: 2

Comments to the Author

This paper represents a novel study of glassy dynamics in ice. It is of broad appeal as it relates to a fundamental issue in physical chemistry, the possible physical states of water. Using x-ray photon correlation spectroscopy the authors observe a transition from a glassy high density liquid state to a low density amorphous phase, and are able to measure the dynamics within these phases. Most interestingly the see a heterodyne signal which indicates interference between static scattering, likely from LDA domains floating in a viscous HDL phase.

This paper should be published, but I would like to recommend the authors address a few clarifications and comments:

1. Page 8, line 7. I am confused by what sort of flow the authors consider to be plausible for the measured heterodyne. They exclude gravitational or lateral flow. Does this mean they expect the flow to not be along a single direction. If so, could they either say that or clarify what they mean? It is also suggestive that the flow lines up with a symmetry direction of 45 degrees. This deserves some comment.

2. Why do the authors consider only HDL and LDA rather than the possibility of LDL phase?

3. p. 9 L 38, Could the authors elaborate on how macroscopic expansion could be a possible source of the heterodyne signal?

4. p. 9, L 59 “the heterodyne signal must be due to a second, static, non-diffusive component.” I’m confused by this statement. Eq. 2 seems to indicate that the heterodyne comes from the interference between two diffusive components (otherwise, why is the amplitude of the oscillation proportional to I1 x I2). Can this be clarified in the text?

Reviewer: 3

Comments to the Author

This paper photon correlation studies of the EHDA to LDA transition. A heterodyne signal is observed and a model presented of a distribution of solid LDA embedded in liquid eHDA. Whilst the paper is well written and the analysis is sound. I have two concerns.

1. The transition temperature of between 110 and 115 K is significantly lower than the 125-128 K reported by Nelmes et al (Nat Phys 2006) for fully expanded eHDA. The samples here were produced by a different route than that used by Nelmes et al (decompression of VHDA) which is known to sometime produce incompletely expanded eHDA and this seems to be the case here. The authors need to either acknowledge this and make it clear that this is an incompletely expanded sample or to repeat the experiment.

2. Even if this is addressed I am not convinced the result is sufficiently broad in its appeal to justify publication in JPCL. It feels to me more like a JCP.

Author's Response to Peer Review Comments:

First of all, we want to thank the reviewers for their hard work and valuable comments. Below is a point-to-point response to the different comments:

Reviewer: 1

Li et al. presented an observation of novel dynamics in amorphous ice supported by heterodyne XPCS. The system itself has been studied by the same team (Perakis et al., 114 (31) 8193 2017, Ref. 4), but the observation of heterodyne is quite intriguing. I would recommend publication of the manuscript if the following concerns are addressed:

1. In the XPCS heterodyne analysis, as documented in Ref. 40 (Ulbrandt et al.) and Lewis et al., Rev. Sci. Instrum. 89, 123902 (2018), Q is treated as a vector instead of a scalar. In the scenario where the velocity is of the same amplitude but in opposite directions (e.g., when the sample is under rheological shear and the x-ray beam shines through the middle of the rheometer), such motion may lead to the oscillation of velocity, as shown in Fig. 4d of the manuscript. This might provide additional information to the physical picture of the dynamics. Have the authors considered fitting the phi-dependent g2 with Eq. 8 in Lewis et al.?

We thank the reviewer for pointing out Lewis's paper. We have added it as reference 44 in the manuscript. We note, that in our original manuscript, equation 2 is the same as equation 10 in the Lewis paper. We also treat Q as a vector when we look at the angular dependence. We found an angular

dependent velocity, where the heterodyning is most pronounced in the direction of the highest velocity. To make this clear, we added the following sentence to the manuscript on page 7 - 8:

> "We additionally calculated the $g_!$ functions at different azimuthal angles f for each temperature and Q as depicted in Fig. 4a, hence now treating Q as a vector."
>
> …
>
> "The angular dependence of the velocity, and therefore also the heterodyning, shows a similar trend in different samples …"

2. Have the authors provided background scattering intensity? Static sample holders or windows could result in heterodyning if the intensity is comparable. Such a scenario is unlikely given that the sample is free-standing and the heterodyning is temperature-dependent (Fig. 1-2), however it would be ideal to clarify this as a sanity check.

Indeed, static windows could result in heterodyning. However, as pointed out by the referee, our sample is free standing in vacuum, without surrounding windows as in our earlier studies. In fact, the whole sample chamber is connected without windows to the vacuum of the beamline to minimize the background signal. Parasitic scattering could only be caused by scattering from the beam-size defining slits. We did measure the empty sample holder (with varying X-ray intensity) as well as a free-standing aerogel sample. We do not find any signature of heterodyning in those datasets. The figure presented below shows the aerogel data. We added this information to the supporting information on page 4:

> "Such an effect was also observed in other studies, as e.g. colloidal gels[7]. Using instead an aerogel as reference, measured with a low X-ray fluence to avoid beamdriven effects (as done here as well), no contrast drop is observed.
> Following the examples[7] where such a drop is observed, the size of the localized motion $r_{"\#\$}$ …"

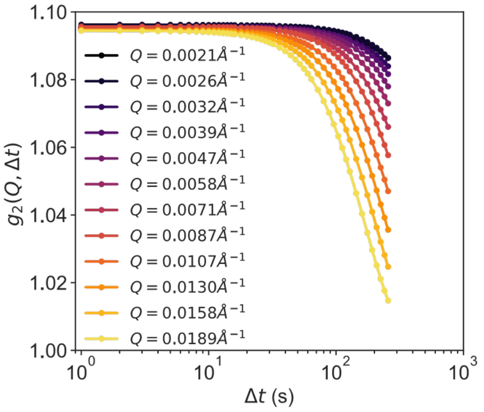

Fig. A: Aerogel reference sample

3. The authors attributed the change of contrast in Fig. 1C to fast dynamics. However, it appears more of an artifact in XPCS analysis as the g2's at different Q are flat at fast time scales and simply offsetted instead of showing a double-stage and impartial decay. Have the authors considered the possible effect

from the intensity variation in the Q-average regions? Some examples include Section 6 and 7 of SI in Xi et al., Soft Matter, 2023, 19, 233 and Fig. S6 in Sheyfer et al., Phys. Rev. Lett. 125 125504 (2020).

The described contrast drop is something we see in many of these samples, as well as in other systems (ref. [13,28,36,37]). However, an aerogel reference sample measured during the same beamtime does not show this contrast drop, but rather a stable contrast of around 10 % throughout the Q-range that has been investigated here (as shown in the figure displayed above). Therefore, we would expect that an offset of the baseline (as discussed in the references the reviewer proposed) would rather lead to an increase of the apparent contrast, while we see a drop of the contrast with respect to the contrast values at low-Q values and the aerogel data. In addition, for the higher temperatures, we can see the baseline within our experimental time window and as the g2-functions show a decreasing contrast value with increasing Q, we argue that this must be a different effect.

Regarding the second part of the question on the intensity variations in the Q-average regions: for the calculation of the g2-functions we used a five-times finer binning of the data for the intensity normalization to minimize the effect of intensity variations within a given q-bin. With regard to intensity variations during the time interval of a measurement, which would also lead to a shift of the baseline, we would like to explain that we always evaluated the total averaged intensity throughout the measurement time. This would look like the two following examples, the plots are taken directly out of our e-log during the experiment (using a Matlab code provided by beamline P10), hence monitored during the experiment. The left figure shows a normal dataset, with three small intensity increases due to the top-up operation of the storage-ring. The figure on the right shows a dataset with an anomalous behavior and an overall intensity decrease and therefore the dataset has not been used for further evaluation.

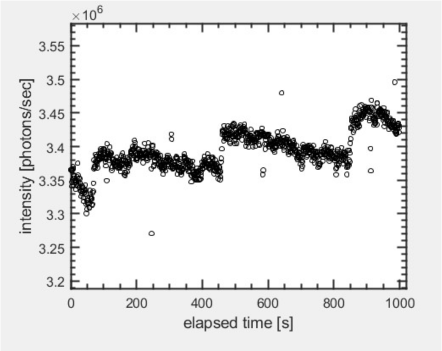

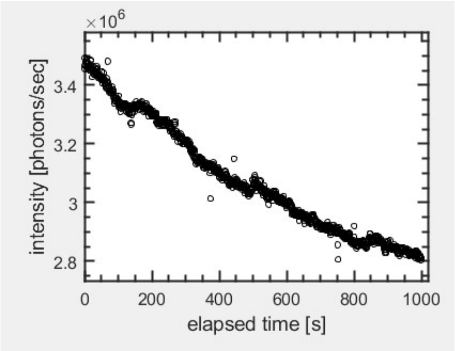

Fig. B: X-ray intensity variations during the time interval of a measurement

4. Fig. 1a shows the 2D detector images with the pixel number as the axis, which does not directly correspond to the Q values and is a mismatch to Fig. 1b. Also, the Q region at which Fig. 1c was calculated did not appear in Fig. 1a and 1b. Is there a particular reason for not showing the 1D and 2D SAXS in those regions?

During the experiment we used 2 different X-ray detectors simultaneously, an Eiger X4M detector located 21.2 m downstream from the sample for the SAXS data and an Eiger 500K detector located 0.147

m from the sample for the WAXS data. The autocorrelation functions displayed in Fig. 1c were calculated from the SAXS detector (Eiger X4M), which offered a sufficient resolution to resolve speckles, while Fig. 1a and 1b plot the WAXS region data using the Eiger 500K detector. The Q-range for the two detectors is limited by their respective size and position. 1D and 2D SAXS data are presented in Fig. S1 and Fig. S9a.

For the WAXS data, the mapping from detector pixels (Fig 1a) to momentum transfers Q (Fig. 1b) is calibrated by measuring a reference powder sample (in our case LaB6). The experimental geometry (spatial relationship between beam, sample and detector) is obtained from a leastsquares fit to control points placed on the Debye-Scherrer rings, as they appear on the detector, using the calibration utilities included in the PyFAI Python library (Ashiotis, G., Deschildre, A., Nawaz, Z., Wright, J. P., Karkoulis, D., Picca, F. E. & Kieffer, J. (2015). J. Appl. Cryst. 48, 510-519). This means, we cannot convert the X-axis of Figure 1a to Q unambiguously, as a constant Q lies along the Debye-Scherrer ring. In order to make the connection clear, we have added this information on page 2 of the supporting information.

> "The XPCS patterns were recorded with an Eiger X4M detector, located 21.2 m downstream from the sample. An Eiger 500K detector was located 147 mm from the sample to record the WAXS patterns. The mapping from detector pixels (Fig 1a) to momentum transfers Q (Fig. 1b) is calibrated by measuring LaB_6 as a reference powder sample. The experimental geometry (spatial relationship between beam, sample, and detector) is obtained from a least-squares fit to selected points placed on the Debye-Scherrer rings, as they appear on the detector, using the calibration utilities included in the PyFAI Python library.[6]"

Additionally, we added "**using two detectors simultaneously**" and "Details can be found in SI." to the figure caption of Fig. 1.

5. The offset of g2 in Fig. 4b is not specified. Also, some g2s have larger overall decay than the others, which is likely caused by the same reason in Comment 3. This may have also caused the crossover between 225 and 203 degrees which looks quite confusing.

The offset in Figure 4b is not specified, as the angular dependent integration results indeed in a shift of the baseline, possibly caused by the very limited number of detector pixels in a Q-bin, which can be used in such a small angular fraction. Contrary to Figure 1c, where we average over all angles, and as explained above, we are sure that the contrast drop is real, this cannot be disentangled in the angular dependent data. We therefore only show a relative comparison of the different angles.

The main message of the analysis is, that the heterodyning appears to be angular dependent, which rules out the possibility of condensed ice as the static component. This is, the larger overall decay at 45° and 225 ° is caused by the less pronounced heterodyning. We assume that this directionality is caused by cracks, grain boundaries and stress relaxation within the sample.

The apparent crossover between 225° and 203° is not a crossover. We apologize for this misunderstanding and slightly changed the offset of 225° to make this visible.

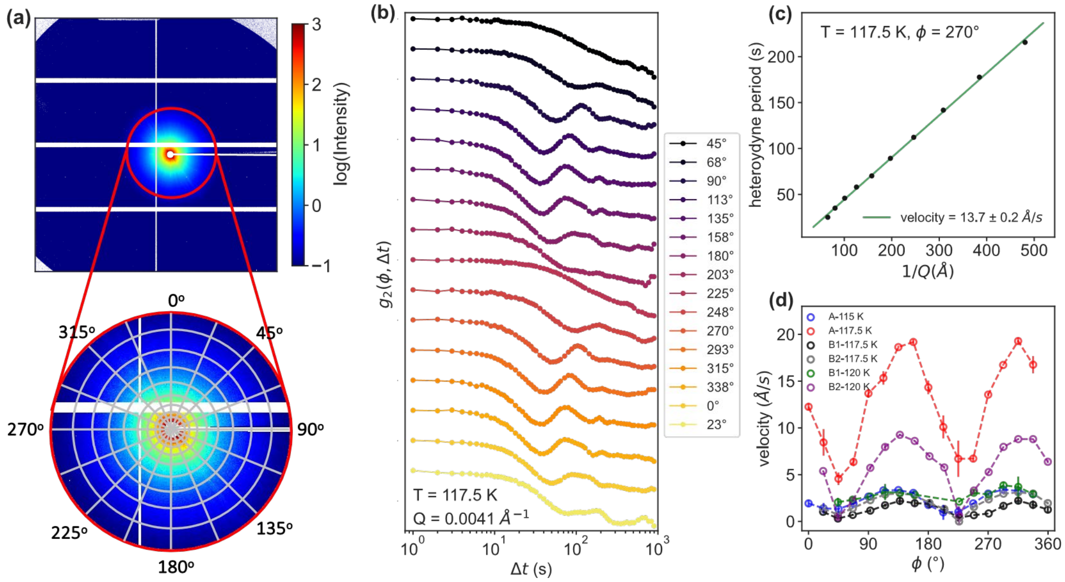

Fig. C: New figure 4 of main manuscript.

Reviewer: 2

This paper represents a novel study of glassy dynamics in ice. It is of broad appeal as it relates to a fundamental issue in physical chemistry, the possible physical states of water. Using x-ray photon correlation spectroscopy the authors observe a transition from a glassy high density liquid state to a low density amorphous phase, and are able to measure the dynamics within these phases. Most interestingly the see a heterodyne signal which indicates interference between static scattering, likely from LDA domains floating in a viscous HDL phase. This paper should be published, but I would like to recommend the authors address a few clarifications and comments:

1. Page 8, line 7. I am confused by what sort of flow the authors consider to be plausible for the measured heterodyne. They exclude gravitational or lateral flow. Does this mean they expect the flow to not be along a single direction. If so, could they either say that or clarify what they mean? It is also suggestive that the flow lines up with a symmetry direction of 45 degrees. This deserves some comment.

Gravitational flow would appear at 180 °, which is not the case here. It is also very unlikely due to the nature of the sample, as the sample is made of one solid piece rather than the powdered sample used in our previous study. In the manuscript on page 8, we describe that the sample, however, contains grain boundaries as well as macroscopic cracks. Cracks and grain boundaries are expected to cause so-called streaks in the detector image, as visible in Figure S9 and S10. That the highest velocity is observed at 45 ° is just a coincidental observation. The streaks are also observed at 45°, which is why we assume there is a direct correlation with the highest velocity, but we cannot provide a full proof as one of the three samples has no streaks. We note that the streaks are masked out for the calculation of g2.

Lastly, the fabrication process during high-pressure amorphization is likely to cause stress inside the sample. We rephrased the text on page 8 as follows:

"The angular dependence of the velocity, and therefore also the heterodyning, shows a similar trend in different samples. A similar behavior of the angular dependent velocity was reported by Dallari et al.[45] when investigating the stress relaxation in repulsive colloidal glasses. As a coincidence, in our measurements streaks appear on the detector (Fig. S9 and S10) at around 45°. Streaks have also been observed in other directions in other samples and temperatures, but here, they appear mostly at this angle. We assume that the streaks are related to grain boundaries or stress.[44]"

2. Why do the authors consider only HDL and LDA rather than the possibility of LDL phase?

We consider LDA formation rather than LDL formation, as the reported glass transition temperature for LDA is at 136 K. We do not see evidence for LDL dynamics in our data, as this goes beyond our analysis. The analysed data focus on processes before the full transition, hence below Tg of LDA. More specifically, at a cryostat temperature of 115 K and 117.5 K, hence an estimated sample temperature of maximum 123 K.

The scenario is still in agreement with a proposed liquid-liquid in water´s phase diagram and also consistent with what was reported in Perakis et al., 114 (31) 8193 2017. The existence of LDL in that study was related to diffusive dynamics after the transition at higher temperatures.

This was already described on page 10:

LDA seeds can act as a heterodyne static component, as its T_g is at a higher temperature (136 K) than for eHDA (115 K). We also added now on page 9:

"Calorimetry and dielectric spectroscopy suggest the glass transition onset temperature to be T_g (eHDA) = 115 K and T_g (LDA) = 136 K, which is consistent with the previous XPCS data on powder samples.[4] The here presented measurements are done below the glass transition temperature of LDA. "

3. p. 9 L 38, Could the authors elaborate on how macroscopic expansion could be a possible source of the heterodyne signal?

We thank the reviewer for this comment. We had mentioned this possibility briefly, but now added the following to the discussion on page 9 and 10.

"In the current work, the XPCS experiments were performed on free-standing amorphous ice films, which allows for a macroscopic expansion with a volume change of 20% during HDA-LDA transition, which is another possible source for the heterodyne signal. We ensured the annealing of the sample for 20 min before each measurement, hence allowing thermally activated motion to relax, after 20 min the WAXS signal remain stable. Still, it is a metastable system, and a fraction of water molecules will – also after equilibration - be displaced when high-density domains expand to low-density regions. The velocity obtained from the heterodyne signal can be related to this displacement

> (expansion). The liquid-like motion of the high-density matrix and the expansion to low-density are two correlated processes which are difficult to disentangle, the heterodyne signal now allows a first estimation."

4. p. 9, L 59 "the heterodyne signal must be due to a second, static, non-diffusive component." I'm confused by this statement. Eq. 2 seems to indicate that the heterodyne comes from the interference between two diffusive components (otherwise, why is the amplitude of the oscillation proportional to I1 x I2). Can this be clarified in the text?

The heterodyne equation does not require necessarily for either of the dynamical processes to be diffusive. The general theory of Photon Correlation Spectroscopy (see e.g. the book "Dynamic light scattering: with applications to chemistry, biology, and physics", B. J. Berne and R. Pecora, Publisher: Dover Pubns 2000) finds that all kinds of density fluctuations lead to intensity fluctuations and are described by correlation functions. Generally these can be described by exponential functions (or distributions of exp function as e.g. KWW). A more general formulation, in which two speckle-patterns "a" and "b" are mixed is: $g_2 - 1 = (I^2_a/I^2_{tot})*|g_1\_a|^2 + I^2_b/I^2_{tot}*|g_1\_b|^2 + 2*(I_a*I_b/I_{tot})*|g_1\_a*g_1\_b|$.

Therefore, the interference term is proportional to the product of both intensity in all cases. Only if G=1/tau, the inverse characteristic time of a correlation functions, follows a Q^2 dependence one can relate this to a diffusive motion. In this sense Eq.2 only describes a sum of correlation functions. Only the further analysis of the behaviour of G can show if a process is diffusive.

To clarify in the text, we deleted "non-diffusive" in the mentioned sentence on page 9 (now page 10). Additionally, we added the following on page 6:

> The heterodyne equation (2) does not require necessarily for either of the dynamical processes to be diffusive. Only if the relaxation rate $\Gamma(Q)$ has a linear relationship with $Q^{!}$, namely $\Gamma(Q) = D_{\%}Q^{!}$, a diffusive motion is present, where $D_{\%}$ represents the Stokes-Einstein diffusion coefficient.[19,36]

Reviewer: 3

This paper photon correlation studies of the EHDA to LDA transition. A heterodyne signal is observed and a model presented of a distribution of solid LDA embedded in liquid eHDA. Whilst the paper is well written and the analysis is sound. I have two concerns.

1. The transition temperature of between 110 and 115 K is significantly lower than the 125-128 K reported by Nelmes et al (Nat Phys 2006) for fully expanded eHDA. The samples here were produced by a different route than that used by Nelmes et al (decompression of VHDA) which is known to sometime produce incompletely expanded eHDA and this seems to be the case here. The authors need to either acknowledge this and make it clear that this is an incompletely expanded sample or to repeat the experiment.

We thank the reviewer for the comment and apologize for the confusion. As pointed out in the supporting information, the given temperatures are measured at the cold head end of the cryostat. Since this is a cold-finger cryostat, rather than a helium jacketed cryostat, a temperature offset to the clamped

ice sample, now free-standing in vacuum, is expected. We have estimated the offset to be around 5 K, as described also in Ladd-Parada et al. (*Environmental Science: Atmospheres* 2022, 2, 1314–1323). This is, the transition takes place at 120 K and is fully transformed at 120 K (T_{cryo}) + 5 K = 125 K (T_{sample}) (Fig. 1). The offset can be explained by the uncertainty in sample temperature, different behavior of the sample in vacuum, as well as – as mentioned by the referee – a not fully expanded eHDA sample due to the different preparation process. In order to make this clear, we added the following to the text on page 4:

> "Complete transition to LDA happens above 120 K (T_{sample} μ 125 K). This is slightly lower than for other studies on eHDA,[30,35] but can be explained by a different sample preparation (see also SI)."

Added to SI, section sample preparation:

> However, the term eHDA here only refers to the preparation pathway [2, 3] , and not to a fully expanded eHDA as demonstrated by Nelmes et al.[4]. How sample preparation and friction can lead to slight shifts in the first diffraction maximum was discussed in literature.[5] Here, in addition the sample is free standing in vacuum, which might result in a shift in thermal stability. Furthermore, the sample temperature cannot be measured directly and is estimated to be +5 K compared to the cryostat temperature.[3]

We also tried to make the temperature offset between cryostat and sample clearer now, throughout the manuscript, e.g.

> $T_{cryostat}$ = 110 K (T_{sample} μ 115 K)

2. Even if this is adressed I am not convinced the result is sufficiently broad in its appeal to justify publication in JPCL. It feels to me more like a JCP.

We hope that the above discussion with the other referees can convince referee 3 that our study is worth being published in JCPL. The different processes which play a role when high-density amorphous ice transforms to low-density are complex, and we believe that our new findings provide a new twist to the long-discussed story. This should also be interesting for other communities, as liquid-liquid transitions emerge to be important in other systems, as e.g. the liquid-liquid transition in proteins, where water might play a major role. XPCS is a relatively young technique, which attracts more and more interest in different communities thanks to the current progress in synchrotron radiation and free-electron laser sources. We believe that the here reported heterodyne signal in a homodyne detection scheme is of broad interest for both the XPCS as well as the DLS community.
